# Energy use and life cycle greenhouse gas emissions of drones for commercial package delivery

**DOI:** 10.1038/s41467-017-02411-5

**Published:** 2018-02-13

**Authors:** Joshuah K. Stolaroff, Constantine Samaras, Emma R. O’Neill, Alia Lubers, Alexandra S. Mitchell, Daniel Ceperley

**Affiliations:** 1 E Program, Global Security, Lawrence Livermore National Laboratory, Livermore, CA 94551 USA; 2 Civil & Environmental Engineering, Carnegie Mellon University, Pittsburgh, PA 15213 USA; 3 Advanced Technology and Systems Division, SRI International, Menlo Park, CA 94025 USA; 4 Chemical and Biological Engineering, University of Colorado at Boulder, Boulder, CO 80309 USA; 5 Present Address: LeoLabs, Inc., PO Box 998, Menlo Park, CA 94026 USA

## Abstract

The use of automated, unmanned aerial vehicles (drones) to deliver commercial packages is poised to become a new industry, significantly shifting energy use in the freight sector. Here we find the current practical range of multi-copters to be about 4 km with current battery technology, requiring a new network of urban warehouses or waystations as support. We show that, although drones consume less energy per package-km than delivery trucks, the additional warehouse energy required and the longer distances traveled by drones per package greatly increase the life-cycle impacts. Still, in most cases examined, the impacts of package delivery by small drone are lower than ground-based delivery. Results suggest that, if carefully deployed, drone-based delivery could reduce greenhouse gas emissions and energy use in the freight sector. To realize the environmental benefits of drone delivery, regulators and firms should focus on minimizing extra warehousing and limiting the size of drones.

## Abstract (web summary)

The use of drones to deliver commercial packages is poised to become a new industry. Here the authors show that replacing truck delivery by drones can reduce greenhouse gas emissions and energy use when the drone size and additional warehousing requirements are limited.

## Introduction

The technological advancement of unmanned aerial vehicles (UAVs), commonly referred to as drones, enables novel applications in industry, government, and research^1^. Drones have long been used in military applications, but the use of drones for commercial package delivery is poised to become a new industry. Several companies are developing programs for package delivery using drones, including Amazon^2^, Google^3^, UPS^4^, and Deutsche Post DHL^5^. Some of these firms have registered patents ranging from new delivery drone designs to flying warehouses^6,7^. Although commercial use is currently limited in the United States and much of the world, the U.S. Federal Aviation Administration and European Aviation Safety Agency are developing regulations to allow more commercial uses^8–10^. Much of the previous criticism of commercial drones has focused on privacy concerns and safety issues, both of which are significant^11,12^. Yet widespread adoption of drones to replace some truck deliveries could reshape the energy system by changing total demand and by shifting a portion of the demand for fuel, for example, from diesel used by trucks to electricity used by drones. Truck transport is responsible for 24% of transportation-related greenhouse gas emissions and comprises 23% of transportation energy use in the USA^13^, hence changes to the industry are important to the environment and the energy system^14^. While power grids are evolving, the scale of environmental benefits from charging drones with electricity depend on the life-cycle environmental characteristics of electricity, vehicle use, battery materials, and enabling infrastructure. Drones are coming to the transportation sector, and stakeholders need to be prepared to encourage positive environmental outcomes during this transition. Understanding these issues can inform public and private decision makers facing energy and environmental choices in the infancy of the commercial drone age.

Previous studies have shown that transport of goods per tonne-km by conventional aircraft is about four times more carbon-intensive than transport by truck, which is in turn about 10 times more carbon-intensive than transport by rail^15^. A delivery system based on drones carrying single packages promises a new class of delivery speed—Amazon has claimed 30 min from time of purchase^2^. If it follows the trend set by other modes, this large increase in speed could come at the cost of another order-of-magnitude increase in energy intensity and carbon emissions. However, small, battery-powered drones can be considerably more efficient than the fossil-fueled vehicles they replace.

Most drones for military and government applications have been larger, fixed-wing (airplane-style), fossil-fueled designs. Fixed-wing aircraft are inherently more energy efficient and can have much longer ranges than rotary wing copters, and this holds true for small drones^1^. However, these fixed-wing drones are physically much larger than copters of comparable payload capacity, they require runways for takeoff and landing, are more constrained in the weight and size of packages they can carry, and can only deliver packages from an altitude, while moving, which is not as suitable for precise urban deliveries. Drone deliveries have also been considered for places with poor road networks, for example delivering supplies to villages in rural Africa^3,16^. This would require longer-range fossil-fueled drones, and fixed-wing drones making airdrops of larger payloads are more plausible for this application. However, drone deliveries are also likely to appear in urban developed markets.

In most prototypes and media depictions so far, drones for package delivery have appeared as four- or eight-rotor, battery-powered copters capable of carrying up to 4.5 kg (10 lbs). The multicopter models currently available typically have flight times of 10–15 min before draining the onboard batteries, leading to very limited delivery ranges. Larger batteries, new battery technologies, and other energy storage methods would affect the range and energy efficiency of these drones. Google has announced a novel hybrid design, capable of both hovering and fixed-wing flight^3^. Although not meant for package delivery, another hybrid prototype has been revealed by Sony^17^. However, to date, information about this type of drone is limited and commercial models are not available.

In addition to the energy efficiency of the drone designs, the impacts of drone delivery depend on the structure of the logistics system in which they are employed. Several visions have been offered, and these include a drone flying directly from the retailer’s warehouse to the consumer, a package transported by multiple drones in relay through a series of waypoint stations^3^, or the drone taking packages from a truck to the consumer and back while the truck travels near a series of delivery locations^5^. The advantage of drone delivery for consumers in developed markets has mainly been represented as speed. Several logistics scenarios can offer near-immediate delivery if coupled with a network of local warehouses of available commercial goods. Thus, the energy use and impacts of any additional product warehousing must also be considered.

Here we develop scenarios for truck and drone delivery to compare impacts among drones and traditional delivery methods. We focus the analysis on copters, by far the better-known and more widely depicted format for package delivery, but also include an initial estimate for a fixed-wing (non-hybrid) drone for comparison. To assess these issues, we develop a flexible energy use model for multi-rotor drones that is calibrated to measurements from representative quadcopter flights. We characterize the life-cycle greenhouse gas (GHG) emissions and energy impacts of commercial package delivery by drone compared with current systems. We show that while drones could consume less energy per package than diesel-powered delivery trucks, the additional warehouse energy required greatly increases life-cycle GHG impacts. Still, in most cases examined, we show the life-cycle GHG emissions and energy use of package delivery by small drone are lower than ground-based delivery. Minimizing extra warehousing and continued reductions in electricity carbon intensity are of critical importance to realizing the environmental benefits of drone delivery.

## Results

### Drone sizes and ranges

To estimate the energy use and environmental impacts of drone delivery, we first model the energy use and performance of multicopter drones. The use case consists of a single drone carrying a single package from warehouse to destination, and then returning empty to the warehouse. We base the analysis on two representative commercial drones: a small quadcopter (3D Robotics’ Iris), designed to carry up to 0.5 kg, and a large octocopter (Turbo Ace’s Infinity 9) designed to carry up to 8.1 kg. These provide useful bounds in the performance of current commercial designs. To estimate the energy used by a drone on a delivery trip, we extend a known analytical model, based on conservation of momentum, of the theoretical minimum power demand by copters. The model includes two empirical parameters: an overall power efficiency, *η* (compared to the theoretical minimum), and effective drag coefficient for the drone body and rotors, *C*_D,body_. These are derived from measurements of 1073 flight segments of the quadcopter in several outdoor test campaigns (see Methods for model details and flight conditions). Parameters thus account for moderate wind (0–7 m/s) at random orientation to the direction of travel.

**Fig. 1:**
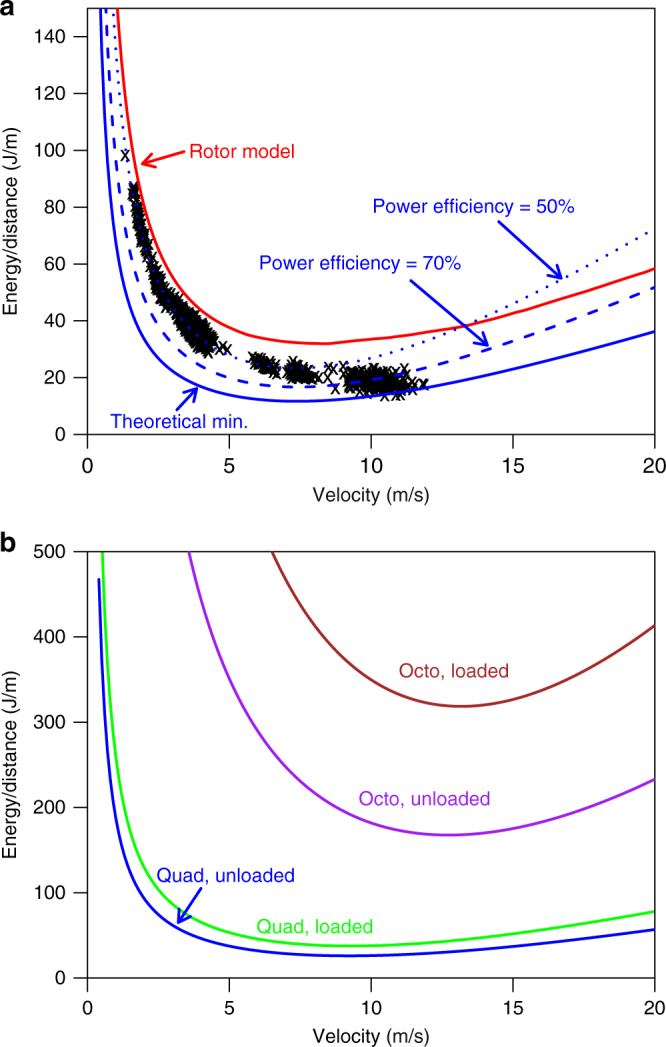
Energy use of a drone per distance traveled at various velocities. **a** Comparison of measured data with several models for the unloaded quadcopter. The theoretical minimum (theoretical min.), represented by the solid blue line, is calculated by Eqs. 6 and 7. The dashed blue line shows the power efficiency curve at 70% and the dotted blue line the power efficiency at 50%. These are calculated by Eq. 8 with the values of *η* (power efficiency) noted. The red line shows results incorporating the manufacturer-supplied rotor properties (see Methods for rotor model). Black crosses denote measured data for 1073 flight segments. **b** Base-case model results for quadcopter (Quad) and octocopter (Octo), with and without a package

The energy used by the drone to deliver a package depends on the speed of travel. Figure 1a shows the measured energy use per distance traveled over a range of velocities for our test unit quadcopter. Comparable results from the analytical model are shown with two different power efficiencies. At low velocities, a power efficiency, *η*, of about 50% fits the data well (also with different payload masses, see Supplementary Figs. 1 and 2). At higher speeds, the power efficiency appears to increase to about 70% (likely due to translational lift, a well-known effect in helicopter dynamics^18^). We use the higher value in further analysis. The effective drag coefficient, *C*_D,body_, also varies with velocity (Supplementary Fig. 3). We use the best-fit value at higher velocities, *C*_D,body_ = 1.5, in further analysis. Figure 1b shows the model results for the quadcopter and octocopter in the two relevant flight conditions (with and without a package).

In selecting the velocity, the operator may choose to minimize energy use (i.e., maximize range) or may choose higher speeds for faster delivery times (and better capital utilization), or lower speeds for safety or other practical considerations. The size of the package also influences the optimum choice of velocity, although the overall contribution of package drag in our base case is small (9 and 7% of roundtrip energy use for the quadcopter and octocopter, respectively). For simplicity, we assume a 10 m/s velocity for further analysis in all cases, which yields power demands close to the modeled optimum (at most 14% higher). Supplementary Tables 1–3 present model parameters and assumptions.

**Fig. 2:**
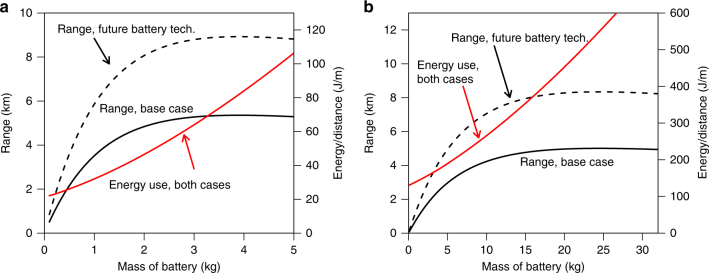
Range and energy use as a function of battery size for model copters. **a** Quadcopter results. **b** Octocopter results. Range (solid black lines) is the sum of a loaded and unloaded trip of the distance shown. The future battery technology (future battery tech.) curves (black dashed lines) reflect batteries with higher energy density than currently available (DOE target for 2022^19^). Red lines show the energy use as a function of battery mass, which is the same for the base case and future battery technology cases. The design battery sizes for the quadcopter and octocopter test models are 0.26 and 4.2 kg, respectively

In our flight tests, the quadcopter flew total distances of 2.6 to 3.7 km on a single charge during the high-speed measurements (5–12 m/s) and 0.6 km to 1.15 km during low speed measurements (<5 m/s), without a package. For the scenarios below, we define range as the farthest distance from a warehouse to which a drone can deliver a package and still return to the warehouse. The test model configuration is thus not suited to package delivery, except at very short ranges (about 1 km). However, increasing the battery size can increase the range. Figure 2 shows the dependence of range and energy use on battery mass. In principle, the range increases with battery size with diminishing returns, up to a maximum where additional battery mass becomes counter-productive. Although the model accounts for the increase in drag due to the presence of the additional battery volume, we find this effect has very little influence on the result compared with the weight of the battery. The maximum range with base-case assumptions is about 5 km for both the quadcopter and octocopter. However, the maximum is achieved with batteries that are impractically large and well beyond the thrust capabilities of the test models, likely imposing problems for flight control, capital cost, and energy efficiency. For further analysis, we choose battery sizes that provide most of the range while still within or near the thrust capabilities of our test units: 1 kg for the quadcopter and 10 kg for the octocopter, providing ranges of about 3.5 and 4.2 km, respectively. These ranges are short compared to the distances traveled by conventional package delivery vehicles and the sizes of most metropolitan areas.

### Powering drones

The choice of battery or energy storage media substantially affects the delivery range and environmental impact of a drone. Lithium polymer (LiPo) batteries for electric motors, and gasoline or glow fuel (a mixture of methanol, oil and nitromethane) for combustion engines are currently used to power drones. To compare, energy densities of relevant energy storage options are shown in Table 1 based on existing and expected battery, hydrogen fuel cell, and popular internal combustion engine technologies and fuels. Practical energy densities are reported for commercially available devices; most need improvement to approach their theoretical values. We include a representative range for hydrogen fuel cells in Table 1 based on estimates of additional hardware necessary to be comparable to a battery. Our base case assumes the energy density of the LiPo batteries used in our test unit, 540 kJ/kg (150 Wh/kg), which is on the high end of current technologies.

**Table 1:** Key characteristics of potential energy storage technologies for drones

Battery chemistry/fuel cell/combustion fuel	Theoretical maximum energy density (Wh per kg/Wh per l)	Practical energy density (Wh per kg/Wh per l)	Proposed maximum # of cycles	Depth of discharge (%)	Representative range^a^(km)
*Metal battery technology*					
Nickel metal hydride	800/1940^71^	80^72^/190^b^	200,000^71^	80^73^	1.8
Zinc-air	700^74^/NA	400^74^/NA	200^73^	80^73^	9.1
Lithium-air	5000/1000^75^	1000^75^/200^b^	1^75^	40^76^	23
*Li-ion battery technology*					
Lithium polymer	890/1440^73^	150/170^77^	300^73^	80^73^	3.5
Lithium iron phosphate (LFP)/carbon	580/2080^78^	130/250^79^	3000^79^	100^79^	3
*Fuel cell technology fuels*					
Hydrogen (200 bar)	33,300/530^80^	16,650^81^/270^b^	9000^c^	100^d^	11
Methanol	5550/4390^80^	2220^81^/1760^b^	9000^e^	100^d^	20
*Hydrocarbon combustion fuels*					
Gasoline	12,400/9100^81^	4710^e^/3450^e^	80,470^f^	100^d^	—
Glow fuel^g^	4310^h^/3930^h^	1640^e^/1500^e^	80,470^f^	100^d^	—

^a^ Representative range is calculated by Eq. 11

^b^ Calculated from density determined from theoretical values

^c^ Calculated from 2500 h maximum lifetime^82^, with a cycle consisting of 16.6 min

^d^ Considering full use of a fuel tank

^e^ Calculated from efficiency of internal combustion engine^83^

^f^ Calculated from 250,000 mile maximum lifetime, with a cycle consisting of 5 km

^g^ Composed of 50% methanol, 30% nitromethane and 20% synthetic oil

^h^ Calculated from mixture characteristics

Figure 2 also shows the calculated range with a future battery technology with 900 kJ/kg (250 Wh/kg), the U.S. Department of Energy target for the specific energy of ground vehicle batteries by the year 2022^19^. We can see from Table 1 that such a target is possible with a variety of battery chemistries, which would enable factor increases in range. We can also see that fuel cell technologies, like combustion fuels, offer significantly higher energy densities and potential ranges compared to current batteries. As electric drones are likely to be the first type of urban delivery drones in widespread use, we focus the primary efforts of this paper on electric drones.

### Comparing emissions

In the near-term, it is likely the use of drones for retail package delivery will only be for short distance, same-day deliveries, and will build upon the existing freight logistics network. Manufacturers produce goods, which are delivered via marine transport (for imports), air, rail, and/or truck to regional warehouses. Once purchased by consumers online, the goods are shipped to local package collection and distribution centers via air transport (for express deliveries) and/or truck, and then shipped along with many other packages by a ground delivery vehicle to the customer’s address. Drone delivery would shift the energy and GHG emissions in this supply chain in two major ways. First, delivery by drone shifts energy use and GHG emissions from the main ground vehicle fuels of diesel, gasoline, and natural gas to the regionally varying sources of electricity used to charge drones. Second, same-day delivery by drone likely requires additional warehouses to store packages in locations close to final customers, so the total energy use by package warehouses could increase. We estimate GHG emissions across three broad delivery pathways. The first is final delivery by medium-duty delivery truck, representing the most common existing logistics pathway. The second pathway is package storage in additional urban warehouses, with final delivery by electricity-powered drone or package delivery vans. The final pathway we assessed is customer pick-up from a retail store or urban warehouse by a passenger vehicle. An illustration of the estimation system boundary used in our model is shown in Supplementary Fig. 4. The total energy and emissions used to deliver packages up to the start of each pathway are assumed to be similar for either ground delivery or drone delivery. The analysis here focuses on the final delivery of the package.

Drone batteries will be charged by the electric grids where they are operating, which differ by region and over time in their fuel mixes and corresponding emissions^20,21^. To account for this variation, we use the U.S. Environmental Protection Agency’s (EPA) estimate of regional, non-baseload GHG emissions in the United States^22^. To bound the main results, we use emissions factors for California (a low-carbon region), the U.S. average, and Missouri (a region with the current highest value). We also report results for drones used in each of the EPA electricity regions and each North American Reliability Council regions as shown in Supplementary Figs. 5 and 6. We use EPA non-baseload power plant fuel mixes and Argonne National Laboratory values^23^ to estimate and include upstream emissions associated with power plant fuels. Because the emissions intensity of the electric grid is likely to decrease over the coming decades, we also include a low-GHG case about half as carbon intensive as California in the sensitivity analysis. We include values for upstream battery manufacturing emissions for drone and electric vehicle pathways (see Methods).

**Fig. 3:**
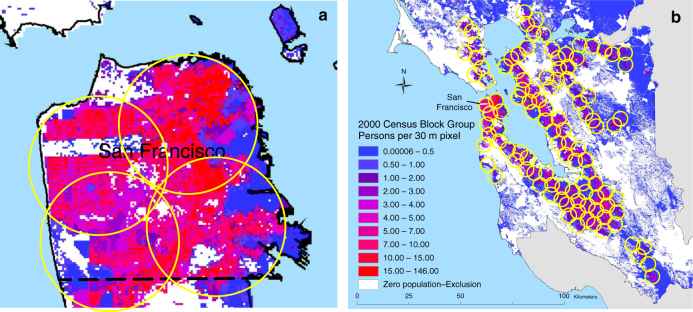
Example coverage of drone delivery systems. **a** coverage with the base-case quadcopter range (3.5 km) in the city of San Francisco, CA, USA, and **b** in the populated areas of the San Francisco Bay Area. Legend indicates population density in the underlying map, which is based on the 2000 U.S. Census^70^. As shown, roughly four local warehouses or waystations (indicated by a yellow circle) would be required to service the small, dense city of San Francisco and up to 112 warehouses or waystations would be required to cover the greater Bay Area

Similar to electric ground vehicles, battery-powered drones benefit from more efficient motors than combustion engines, and the ability to shift urban tailpipe emissions and associated impacts to more remote power plants^24,25^. Yet because of their limited range, the use of drones for on-demand package delivery likely requires additional infrastructure in the form of urban warehouses. These distributed warehouses would need to store a range of products to enable rapid delivery to consumers, increasing the total inventory and floor space required. Another possibility is combining warehouses with urban waystations, where packages could be relayed to fully charged drones, thereby increasing total drone range. In either case, many new warehouses or waystations would be required to support a drone-based delivery system. As an example, Fig. 3 shows a hypothetical coverage map for the city of San Francisco and for the populated areas of the greater San Francisco Bay Area. About four urban warehouses would be needed to provide drone delivery service to the small, dense city of San Francisco, CA with drones capable of a 3.5 km range. Dozens of warehouses and waystations would be required to service a metropolitan area (we estimate 112 for the San Francisco Bay Area). Increasing drone range can significantly reduce the number of needed nodes. For example, in a generic, medium-sized, square city, doubling the drone range reduces the number of required nodes by a factor of 3.5 (see Supplementary Fig. 7 and Methods).

If drones are restricted to designated airways, the warehousing requirements would be higher. On the other hand, modifying existing retail locations to store goods and charge drones could reduce some warehouse impacts. Overall, the warehouse needs represent a large source of uncertainty in estimating emissions from drone delivery. All product delivery pathways require electricity and natural gas to light, operate, heat, and cool package warehouses and distribution centers^26,27^, but any additional warehousing space needed to enable drone delivery results in additional energy use. We quantify this warehousing penalty as a multiple of existing warehouse needs for package delivery. We include emissions for warehouse energy for all pathways, and include emissions for one additional warehouse stage for the drone and delivery van pathways. Although the specific warehouse needs and impacts per package are uncertain, it is likely that the total warehouse area used in a future with widespread drone delivery is higher than the status quo. Although we did not specifically evaluate flying warehouses as patented by Amazon^7^, we consider different ground warehousing multipliers in the sensitivity analysis, which enables a comparison across a range of warehouse energy assumptions.

**Fig. 4:**
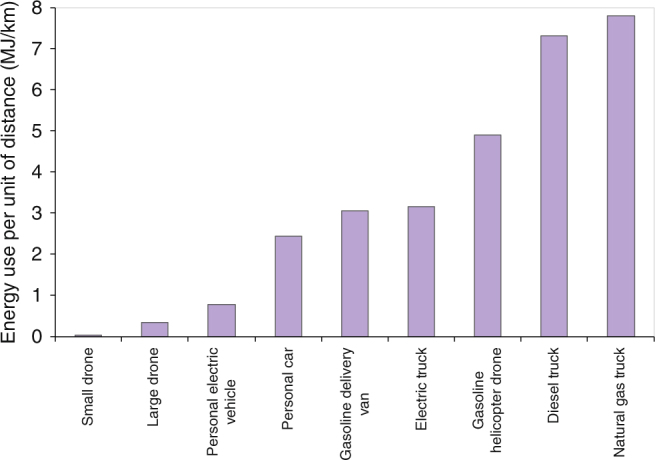
Energy required per km of travel for individual package delivery vehicles. Electric drone and electric vehicle energy use shown includes losses in transmission, distribution, and charging. Electrical conversion losses at the power plant not shown, but are included for the emissions analysis. Energy use per package delivery is dependent on number of packages per km, in addition to a range of vehicle and route characteristics. Life-cycle energy and emissions per package additionally include upstream impacts from batteries and fuels, as well as increased warehouse energy requirements

**Fig. 5:**
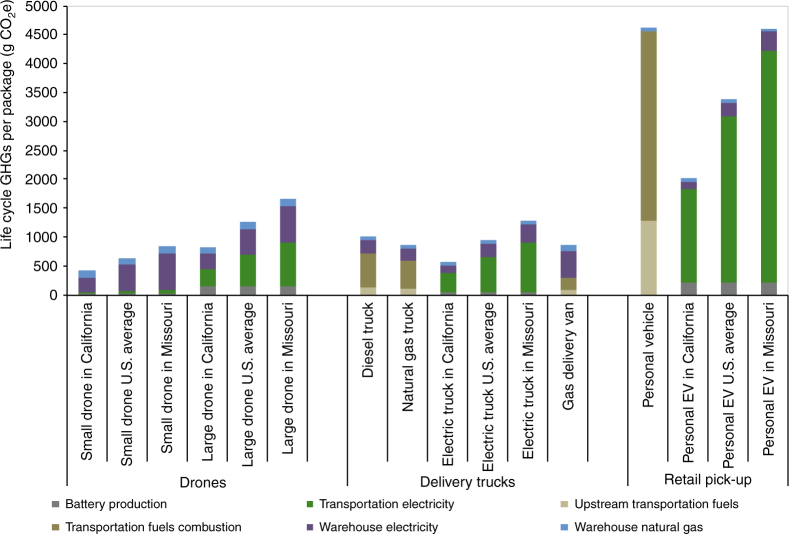
Comparison of life-cycle greenhouse gas emissions per package delivered for drone and ground vehicle pathways under base case assumptions. The analysis focuses on the final delivery of the package, after the package is delivered to the regional warehouse. Emissions from battery and fuels production, as well as fuels combustion and electricity production required for transportation and warehousing, are included. The range of regional greenhouse gas (GHG) intensities of electricity in the U.S. is represented by comparing results from low-carbon California to relatively high-carbon Missouri. Additional warehousing requirements for drone and van pathways are included. The results show that small quadcopter drones across all U.S. regions have lower life-cycle GHG emissions than conventional delivery trucks powered by diesel and natural gas, electric vehicle (EV) trucks in most regions, and gasoline-powered vans. Large octocopter drones are shown to have lower GHG emissions than diesel and natural gas vehicles only when charged with low-carbon electricity. Both small drones and large drones are shown to have lower GHG emissions than use of a personal vehicle to pick-up a single package. Numerical values of these results are presented in Supplementary Tables 13–17

**Fig. 6:**
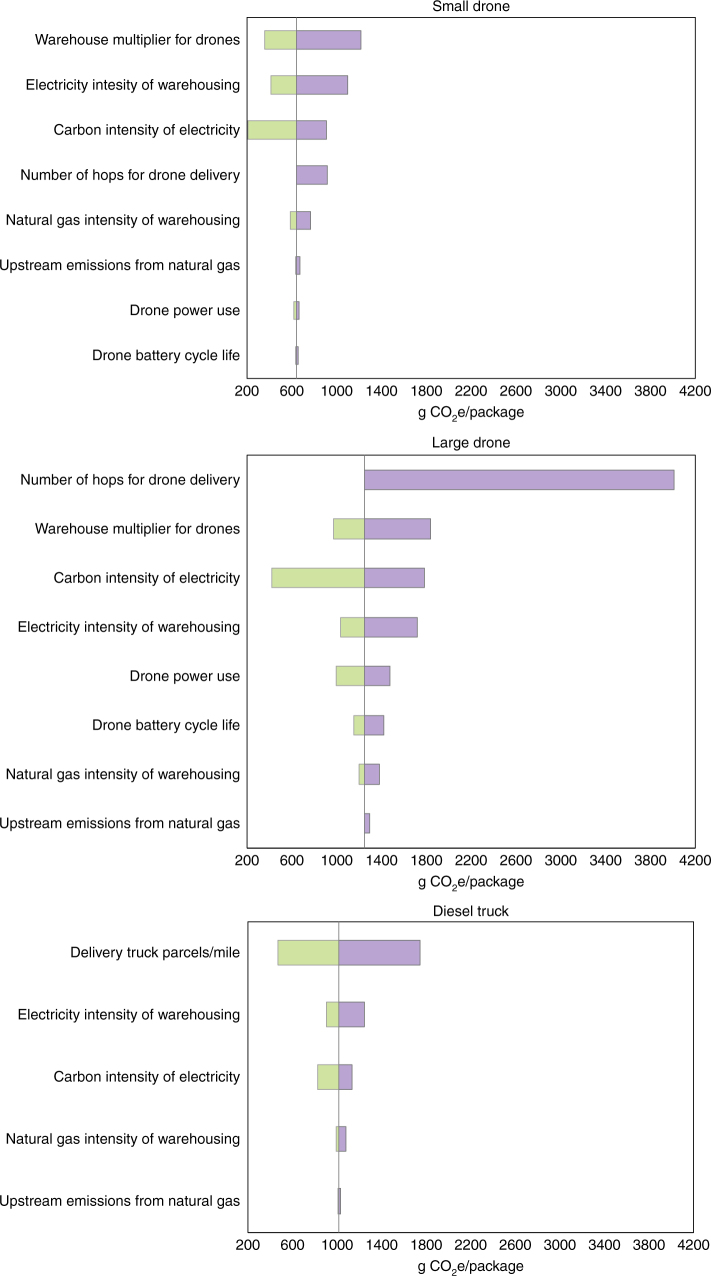
Sensitivity analysis. Greenhouse gas emissions per package delivered for the quadcopter, octocopter, and diesel truck under the parameters listed in Table 2. Vertical lines mark the base case result for each mode. Green bars show the decrease in emissions and purple bars show the increase in emissions given parameters Table 2

Because of their small size, when solely comparing the energy use required per km of distance traveled, we find that electric drones are far more efficient than trucks, vans, larger gasoline drones, and passenger cars. The results are shown in Fig. 4. For cases comparing a drone and a vehicle carrying a single package over similar distances, for example a customer picking up a package from a retail store, the drone is clearly a lower-impact solution. However, delivery trucks carry many packages, delivering on average 0.94 packages per km (1.51 per mile), and their impacts need to be allocated per package. Our base case estimate of energy per package shows drones, electric and gasoline vans, and electric trucks with the lowest values (see Supplementary Table 4). In turn, the greenhouse gas impacts per unit of energy vary by type of fuel. The direct and life-cycle emissions factors are shown in Supplementary Table 5. Taking these factors together with the warehousing impacts described above, we estimate the life-cycle energy use and greenhouse gas emissions (in CO_2_-equivalents) per package delivered by drone, truck (diesel, natural gas, and electric), van, and picked up in a passenger car (gasoline and electricity). The main results are shown in Fig. 5. Electric delivery van results across a range of carbon intensities are shown in Supplementary Fig. 8, and a sensitivity analysis showing how the results change under alternate assumptions is presented in Fig. 6. The assumptions used in the sensitivity analysis are shown in Table 2.

**Table 2:** Parameter values used in the sensitivity analysis

Parameter	Best case	Base case	High case
Number of hops for drone delivery^a^	—	1	4
Carbon intensity of electricity (g CO_2_e/(kW·h)	100	654	1000
Warehouse multiplier for drones and vans^b^	1	2	4
Electricity intensity of warehousing (kW·h/package)	0.175	0.35	0.70
Drone power use (% of base case)	50	100	140
Drone battery cycle life	1000	300	150
Upstream emissions of natural gas^c^ (g CO_2_e/MJ)	10.3	20.1/13.4	29.5
Natural gas intensity of warehousing (MJ/package)	0.475	0.95	1.9
Delivery truck parcels delivered per mile	6.04	1.51	0.76

^a^ Hops are the number of segments each package travels by drone. More than one hop would represent a scenario built on waystations where packages are passed from one drone to another

^b^ The impacts of warehousing in the drone and van scenario, as a multiple of warehousing impacts in the current logistics network

^c^ base case upstream natural gas emissions for CNG/stationary combustion are from Argonne National Laboratory^23^ for consistency. Low (5%) and high (95%) values are from ref. ^40^

In the base case, delivery of a small (0.5 kg) package with the small quadrotor drone has lower impacts than delivery by diesel truck, ranging from a 54% reduction in GHGs in California, to a 23% reduction in Missouri. Small drones also have lower impacts across every other delivery method in the same geographical region: natural gas trucks, electric trucks, gasoline vans, and electric vans. Results for the larger octocopter are mixed and less favorable for drones. In the base case, delivery of a medium-sized (8 kg) package has 9% lower GHGs than delivery by truck in California, is about 24% higher than delivery trucks for the U.S. average electricity mix, and has 50% higher GHGs than truck delivery in Missouri, which has a carbon-intensive electricity grid. Large drones only have lower impacts than the other delivery modes when charged with relatively low-carbon electricity, and still in low-carbon regions such as California, electric trucks and electric vans are better than large drones. Both small drones and large drones have lower GHGs per package than using a personal gasoline or electric car for a single item shopping trip, as shown in Fig. 5.

These results are sensitive to a number of uncertain parameters, as summarized in Fig. 6. For small drones, the number of warehouses needed, the electricity intensity of warehousing, and the carbon intensity of electricity dominates the total impacts. Reducing the number of warehouses, increasing their energy efficiency, or increasing the range of small drones through more energy-dense storage technologies or waystations could reduce overall impacts by reducing warehousing demands.

For the larger and heavier octocopter, the electricity used for flying is more important, and is approximately equivalent to impacts of warehousing in the base case. Therefore, increasing the range through waystations is likely to increase emissions from electricity use more than the corresponding savings in warehousing demand in the near-term. Because of the importance of electricity used to power the octocopter, charging with low-carbon electricity of 200 g CO_2_e/kWh can reduce delivered package GHGs by 37% compared to diesel trucks.

For the truck delivery pathways, the most important parameter is the delivered packages per unit of distance. This is a function of urban density, geography, logistics, and customer orders. Our base case uses average numbers from UPS (Methods), and while deliveries in dense cities could be much higher, additional uncertainties, such as allocating packages across residential, commercial, and institutional customers, pickups, and various package weights, would affect the results. We test a best case where delivery trucks achieve four times as many deliveries per mile as our base case, and only then would diesel trucks start to approach the GHG impacts of small drones in California. However, as technology improves in the future, delivery trucks and vans will get more efficient, reducing emissions per package. Overall efficiency of the logistics system may also improve, due to factors including greater application of information technology and automation, growing horizontal cooperation among companies, or standardization of shipping containers^28,29^. However, multi-rotor drones are already close to the limits of efficiency, and any benefits of improved logistics are likely to accrue upstream of drone operation. Technological advances are more likely to improve energy storage technologies, allowing the drones to go farther and carry heavier packages, ultimately leading to higher emissions per package. Over time then, large drone delivery could become the progressively worse option for package delivery. A major exception to this case is if electricity generation decarbonizes faster than delivery vehicles. If charged with low-carbon electricity, drones offer a distinct advantage over diesel and CNG delivery trucks or vans. In recent years in the U.S., transportation and electricity production have both modestly decreased in carbon intensity^30^.

Another possibility for drones to become more attractive is if hybrid hovering/fixed-wing drones become commercially viable and have significantly improved energy efficiency over copters. As discussed above, prototype hybrids have appeared, but information is currently too limited to evaluate these designs. However, we can fairly expect the performance of a hybrid to fall between that of a copter and a classic fixed-wing sized for a similar payload. Using manufacturer data and measurements from a short flight campaign, we measured the energy efficiency of flight for a battery-powered, fixed-wing drone (3D Robotics Aero-M) with a payload capacity similar to the quadcopter test unit. We find the fixed-wing uses about half the energy per distance traveled as the quadcopter. This is broadly consistent with the well-known efficiency advantage of fixed-wing aircraft over copters^18,31^.

There are several reasons to expect that hybrids will not match efficiency of classic fixed-wings. For example, hybrid motors and rotors must be sized to support the full weight of the vehicle, adding weight and drag. Also, the efficiency of fixed-wings is directly related to wingspan and wing area, which may be limited for safety and maneuverability in urban environments (the quadcopter test model measures roughly 0.5 by 0.6 m, whereas the comparable fixed-wing test model spans 1.3 by 1.9 m). However, the potential for improved energy efficiency through hybrid designs is clear. A scenario where drone energy use is reduced by half is shown in the best case drone power use scenario in Fig. 6. Especially for the larger drone, emissions are reduced in this case, which start to have lower impacts than diesel trucks under the U.S. average carbon intensity of electricity. The range of the drone could be much longer in this case, easing warehouse demand as well, although that is not captured in this calculation.

Although drone-based package delivery faces many technological challenges, safety issues, regulatory concerns, and system uncertainties, in this initial estimate we find significant promise in the use of drones to reduce energy use and greenhouse gas emissions in the freight sector compared with traditional pathways. We focus initially on the United States, where the large variation in regional electricity mixes provides important comparisons and can inform policymakers and stakeholders (the carbon intensity of many countries and international regions fall within the range of the values assessed here—see Supplementary Fig. 9).

There are plausible scenarios where drones lead to overall higher energy use and greenhouse gas emissions compared to ground vehicles. These include higher than expected warehousing space and energy needs, as well as continued improvement in ground vehicle energy and logistics efficiencies. Contrary to most energy technologies, future technological improvement in drones may increase energy use per package, because better energy storage will allow drones to fly further with heavier loads. To realize any environmental benefits from the use of drones, regulators and firms must consider the system-wide demand for additional warehousing, the size and efficiency of the drones, and the source of electricity. In particular, the focus of drones should be on light packages, with heavier packages left for ground vehicles. Finally, it’s clear that the continued reduction in the carbon intensity of the electricity system, coupled with energy efficiency improvements in associated commercial buildings, are essential to realize the potential environmental benefits of freight delivery by drones.

## Methods

### Drone energy use model

Drones expend energy to fight gravity and to counter drag forces due to forward motion and wind. The drone’s control software adjusts the speed of each rotor to achieve the thrust and pitch necessary to stay aloft and travel forward at the desired velocity. On average, the thrusts from each of the rotors are roughly equal and together exactly balance gravity and drag forces. We can then find the total required thrust by:

1 $$T = \left( {m_{{\mathrm{body}}} + m_{{\mathrm{batt}}} + m_{{\mathrm{package}}}} \right)g + F_{{\mathrm{drag}}},$$

where *m*_body_, *m*_batt_, and *m*_package_ are the masses of the drone body, battery, and package (if present), *g* is the gravitational constant, and *F*_drag_ is the total drag force. Similarly, for steady flight, the pitch angle, *α*, can be calculated by:

2 $$\alpha = {\mathrm{tan}}^{ - 1}\left( {\frac{{F_{{\mathrm{drag}}}}}{{\left( {m_{{\mathrm{body}}} + m_{{\mathrm{batt}}} + m_{{\mathrm{package}}}} \right)g}}} \right).$$

The drag force can be estimated piecewise by the formula:

3 $$F_{{\mathrm{drag}}} = \mathop {\sum}\limits_i {\frac{1}{2}\rho v_{\rm {a}}^2C_{{\mathrm{D}}i}A_i},$$

where *v*_a_ is the air speed, *ρ* is the density of air, and *C*_D*i*_ and *A*_*i*_ are the drag coefficient and projected area (perpendicular to *v*) of the *i*th component. We find it useful to consider the drag in three components: the drone body, the battery pack, and the package. Drag coefficients for the latter two are taken from the literature for appropriate geometries. The drag coefficient for the quadrotor drone is determined empirically using the drone’s onboard pitch measurement while flying at various velocities:

4 $$C_{\rm {D}} = \frac{{2m_{{\mathrm{body}}}g \cdot {\mathrm{tan}}(\alpha )}}{{\rho v_{\rm {a}}^2A_{{\mathrm{body}}}}}.$$

The same *C*_D_ is also applied to the octocopter body. The values used are given in Supplementary Table 1.

With the total thrust from Eq. 1, we calculate the power required for steady flight. The theoretical minimum power depends on the area swept by the rotors. In general, larger propellers are more efficient because the larger swept area allows them to achieve a given thrust at lower air velocity. However, they are less responsive (because they have greater inertia) and more dangerous (because they carry more kinetic energy). In addition, rotors must be spaced not to interfere with each other. These factors limit the rotor size.

For *n* rotors of diameter *D*, the theoretical minimum power to hover is^32^:

5 $$P_{{\mathrm{min,hover}}} = \frac{{T^{3/2}}}{{\sqrt {\frac{1}{2}\pi nD^2\rho } }}.$$

When the drone moves at significant velocity or in significant wind, the minimum power requirement changes somewhat depending on the air speed and incident angle. For example, when the drone has substantial forward pitch and forward velocity, the rotors must spin faster to achieve a given thrust, because they must accelerate the air faster than its incoming velocity. The minimum power with forward motion can also be calculated from conservation of momentum. Adapting from Hoffman et al.^33^, the power is given by:

6 $$P_{{\mathrm{min}}} = T(v\,{\mathrm{sin}}\alpha + v_{\mathrm{i}}),$$

where *v*_i_ is the induced velocity required for a given thrust and can be found by the solution to the implicit equation:

7 $$v_{\mathrm{i}} = \frac{{2{{T}}}}{{\pi nD^2\rho \sqrt {\left( {v\,{\mathrm{cos}}\,\alpha } \right)^2 + \left( {v\,{\mathrm{sin}}\,\alpha + v_{\mathrm{i}}} \right)^2} }}.$$

We correct the theoretical minimum power by the overall power efficiency of the drone, *η*, to get expended power:

8 $$P = P_{{\mathrm{min}}}/\eta.$$

We determine *η* empirically for the quadcopter model and apply the same factor to the octocopter estimates. Alternately, the velocity-dependent power–thrust relationship for a specific propeller can be measured empirically or estimated with a computational fluid dynamics (CFD) simulation. The relationship is available from the manufacturer in the form of a lookup table for the propellers in our quadcopter test model^34^. We include the CFD result in Fig. 1 and Supplementary Fig. 1 for comparison.

To calculate the energy efficiency of travel, *e*, we divide power consumption by the average ground speed of the drone:

9 $$e = P/v.$$

We find the energy consumed for a delivery trip of one-way distance *d* by:

10 $$E = \left( {e_{{\mathrm{loaded}}} + e_{{\mathrm{unloaded}}}} \right)d,$$

where power and velocity (which we assume are constant for each leg) are evaluated with the package present to give *e*_loaded_ (for the outbound trip) and without the package to give *e*_unloaded_ (for the return trip).

We calculate the range of a drone, *R*, by:

11 $$R = \frac{{m_{{\mathrm{batt}}}s_{{\mathrm{batt}}}\delta }}{{\left( {e_{{\mathrm{loaded}}} + e_{{\mathrm{unloaded}}}} \right)f}},$$

where *s*_batt_ is the specific energy of the battery (energy capacity per mass), *δ* is the depth of discharge (the fraction of energy capacity designed to be spent on a typical cycle), and *f* is a safety factor to reserve energy for unusual conditions. Note that *P* and *v* depend implicitly on *m*_batt_ because of its contribution to the drone mass. In practice, batteries will have to be oversized to give a margin of safety accounting for variations in conditions and drone performance. On the basis of the variation in flight times that we observed on the test unit, we assume *f* = 1.2. In emergency situations, the option of discharging the battery below its optimal depth of discharge also provides a margin of safety. However, in very windy conditions, especially in the case of a strong and steady crosswind, the drone’s range will be greatly reduced or it may become unstable (in many locations, winds can reach similar speeds to the drone’s air speed). A summary of parameter values used in the base case energy model is given in Supplementary Table 1.

### Drone flight conditions, data collection, and model implementation

Measurements from several flight campaigns with a quadrotor drone were used to validate and calibrate the energy use model. We flew the quadcopter back and forth between programmed waypoints. To obtain data from flight path segments, where the quadcopter was flying at a consistent velocity, we omitted data during the periods when the quadcopter was turning, slowing, or accelerating. There were two sites for testing. One of them required the distance between waypoints to be limited to 10.4 m, but the second site allowed for longer flight paths of 66.1 m, allowing measurements at higher velocities. Our experimental set-up included a weather station that recorded the wind direction and magnitude while we were flying. Wind speed ranged from 0 to 3 m/s (0 to 7 mph) at the first site and 2 to 7 m/s (4 to 15 mph) at the second. On average, wind direction was oriented at random with respect to the flight paths. During different tests, the programmed velocity and acceleration of the quadcopter was varied to examine the energy efficiency at different speeds. We also performed tests with payloads increasing from 0 to 384 g to investigate the relationship between energy use and payload weight. Depending on the flight test parameters, the quadcopter was able to complete between 19 and 74 segments on a battery charge. The onboard autopilot system recorded GPS data, onboard voltage and current, the pitch, roll, and yaw of the copter, and other information. These data were post-processed in Matlab to compute the power drained by the copter, the distance and time traveled, and the drag coefficient. These results were then imported into R for comparison with analytical and manufacturer rotor models and plotting. The analytical and rotor models were implemented in R^35^.

Supplementary Fig. 1 shows the theoretical power vs. thrust relationship for the quadrotor test model. Comparative data shown were measured by affixing a variety of weights and flying at low speed (~2 m/s). We see that the quadrotor operates at about two times the theoretical minimum energy (the best-fit power efficiency is 0.53). This compares well with typical power efficiencies reported for similar propellers^36^. The rotor model overestimates the power consumption slightly.

We performed quadrotor test flights with nine different batteries. The flight times varied depending on payload, programmed speed, and wind conditions. As we would expect, flight times were shorter when the quadrotor carried heavier payloads: flights lasted for 12 min with 0 payload weight, and were as short as 8 min for the full 384 g payload. Flight length extended a little with speed at the first site, and those flights had durations between 11 and 12 min. For the longer distance flights at high speed, there were significantly higher winds and flights lasted between 7 and 9 min. Due to the changing test conditions, there are not sufficient data to formally analyze the variability in flight times, however it is apparent that the variation in flight time per battery charge is substantially less that the variability in energy use on a given flight segment, as seen in Fig. 1a. This is the basis for our assumption of the safety factor, *f* = 1.2.

### Rotor model

For reference, we adapted parameters provided by the rotor manufacturer based on a CFD model to compare with other results^34^. The predicted power and thrust of a rotor is given at various rotational velocities and incident air velocities. However, the parameters do not account for pitch. To calculate the effective incident air velocity, we take the component of air speed perpendicular to the rotor:

12 $$v_{{\mathrm{eff}}} = v{\kern 1pt} {\mathrm{sin}}\left( \alpha \right),$$

where *v* and *α* are the air speed and pitch angle of the drone, as calculated by Eqs. 1–3. The manufacturer-provided parameters are then interpolated using the required thrust and effective velocity to calculate power requirements in various conditions. Results are shown in Fig. 1a and Supplementary Fig. 1. The CFD model gives results of the same order as measured data, but over-predicts power demand at all velocities.

### Energy storage options and drone range

The range of a drone depends strongly on the energy density and specific energy of the battery or fuel it carries. The Lithium polymer battery technology used in the test models and many commercial drones leads to a very short range compared to ground-based delivery vehicles. The representative range for batteries is calculated using Eq. 11 using the quadcopter parameter values in Supplementary Table 1 and the practical energy density and depth of discharge shown. To assess the potential for improvements in drone range and subsequent effects on energy efficiency, we performed a review of battery technologies and hydrocarbon fuels. The results are compiled in Supplementary Table 2, with a shortened version presented in Table 1. The number of charging cycles for drones will affect how often energy storage/generation devices or components need to be replaced. The safe depth of discharge must also be considered, since it relates directly to useable energy density. Additional considerations, such as stability, maximum discharge power and charging rate, are important for vehicle applications^37^, and thus not all chemistries will be available to drones.

Unlike batteries, other components such as fuel tanks and hardware are necessary to facilitate the function of both fuel cells and combustion engines (this weight is not included in the energy densities shown). To compare the performance of fuel cells and combustion fuels to batteries, the added volume and weight must be considered. Table 1 and Supplementary Table 2 give representative ranges for hypothetical drones driven by hydrogen or methanol fuel cells. The key assumptions to calculate the effective energy density of the systems are shown in Supplementary Table 3. Even with the added equipment weight, there is potential for substantially increased range using fuel cells.

Engines used in combusting fuels are generally much heavier than electric motors. Therefore, the practical energy density of combustion fuels will likely be lower than the values in the table when considering the added weights of the motors and added weight of a fuel tank. However, even when considering hardware, combustion fuels are the most energy dense of the technologies we examined, which is why they are used in current large drones.

Advantages to using batteries and fuel cells in drones are higher energy efficiency than combustion, lower noise, no air emissions at the point of use, and possible reduced greenhouse gas impacts via low-carbon electricity storage in batteries or hydrogen produced via low-carbon methods. Cost-efficient use of grid resources is possible if batteries are charged or hydrogen is generated by electricity from the grid during off-peak hours, and then used in drones during peak hours.

### Energy use estimate for a gasoline helicopter drone and a fixed-wing drone

We included parameters for a gasoline-powered drone, which are adapted from the Yamaha RMAX, a helicopter-style drone, currently used in agricultural applications, with a maximum load capacity of 28 kg^38^. As electric drones are likely to be the first type of urban delivery drones in widespread use, we focus the efforts of this paper on electric drones.

Although copters are the focus of this analysis, we also provide an estimate of energy use for a fixed-wing drone for rough comparison. The Aero-M by 3D Robotics is a battery-powered, fixed-wing drone with a payload capacity (0.5 kg) similar to our quadcopter test unit and, incidentally, the same control software. Manufacturer specifications indicate a flight time of 40 min, and maximum and minimum airspeeds of 25 and 9.8 m/s. The specified battery capacity converts to 320 kJ. Assuming an 80% maximum discharge and average flight speed of 17.4 m/s, we calculate an expected energy efficiency of flight of 6 J/m.

To test this specification, we flew a single campaign with the fixed-wing drone and measured energy use and distance traveled. Energy use was calculated by the degree of battery discharge while distance and velocity were calculated by similar methods as for the quadcopter. The flight was performed outdoors in moderate wind conditions at an average ground speed of 13.5 m/s. The resulting energy efficiency of travel was 10 J/m. This should be taken as a rough value, since it is based on a single campaign, however it is consistent with the difference between specified and measured performance that we observed for the quadcopter. Comparing with Fig. 1a, we see that the fixed-wing is roughly twice as efficient as the quadcopter, which clusters around 20 J/m at higher velocities.

This value is roughly consistent with known data for passenger aircraft. Although not directly proportional to energy use, comparing the lift-to-drag ratios of aircraft can provide some guidance about the relative energy use of aircraft of similar weight. The factor-of-2 difference between the copter and fixed-wing drone is on the order of what we would expect comparing the lift-to-drag ratios of passenger helicopters and small planes. For example, a typical ratio for a helicopter is 4.5, whereas the ratio for the 4-seat Cessna 172 is 9. Some aircraft are considerably more efficient, for example the Boeing 747 has a ratio of 17.7, suggesting at least the potential for fixed-wing drones to outperform copters by a wider margin^31^.

### Ground vehicle parameters

For final delivery of packages with a medium-duty truck, we developed a bottom-up energy and emissions model informed by literature values and Argonne National Laboratory’s GREET model^23^ and validated it against top-down sustainability reporting from United Parcel Service, Inc. (UPS)’s 2015 Corporate Sustainability report^39^. We model total energy use of a ground vehicle per package delivered, thereby eliminating the need to estimate exact street routes. Because our functional unit is 1 package delivered, we also do not distribute impacts across the distribution of package weights on a delivery truck. We started with fuel energy content and vehicle efficiency values for Class 4 package delivery vehicles, as well as for light-duty vans and light-duty cars as shown in Supplementary Table 4. The product of the energy content of the fuel and the fuel consumption per mile of travel yields the direct energy use per mile of travel for each of these vehicles.

Tong et al.^40^ estimates that the energy efficiency for Class 4 parcel delivery trucks is 7.3 MJ/km (11.5 mpg) for diesel trucks, 7.8 MJ/km (10.8 mpgde) for compressed natural gas (CNG) trucks, and 2.44 MJ/km (34.5 mpgde) for electric (EV) trucks. Hence, each 100 miles (161 km) of travel at these efficiencies uses 1178 MJ for diesel trucks, 1255 MJ for CNG trucks, and 393 MJ of final energy for EV trucks (excluding efficiency losses at the power plant). As with all rated fuel economy values, actual observed energy use values would vary with driving patterns, traffic, terrain, vehicle weight, age of the vehicles, and other parameters. UPS reported that route optimization and other efficiencies led to a decrease of 23 million miles traveled and a fuel savings of 2.6 million gallons. Dividing those reported numbers only yields 8.9 mpg. Yet, we use the original reviewed literature values as near-term thresholds and also include the bounds of uncertainty to account for near-term improvements to measured fuel economies in these vehicles.

To obtain an energy consumption value for the functional unit of 1 package delivered, the energy used by the vehicle has to be normalized for the number of packages the vehicle is carrying and delivering per km. UPS states^39^ that ground package trucks generally travel about 100 miles/day and average 1.51 stops per mile (161 km with 0.94 stops/km), which provides a base case of 151 packages delivered per day. We note that some of the stops reported by UPS could include pickups only, or could include the delivery of multiple packages to a single stop, which would make 151 packages delivered to individual addresses per day a generous average estimate. Yet, in urban areas packages per mile could be higher. This number of packages delivered per total distance traveled in a day is a key parameter that is explored in the sensitivity analysis. For the sensitivity analysis, we use a best case value of a factor of four higher deliveries per mile than the base, and a worst-case value a factor of two lower. A Class 4 diesel truck that travels 100 miles and delivers 151 packages requires 8.7 gallons, or 17.4 packages per gallon, or 7.8 MJ per package. This estimate is similar in magnitude to an earlier estimate by Weber et al.^27^ of last-mile energy use for commercial package delivery. For U.S. domestic packages, UPS reports a normalized energy intensity of 26.65 MJ per package, however these values include all stationary and fuel energy used by UPS. Taking the percentage of energy from diesel to estimate the energy intensity of final package delivery, as done in Weber et al.^27^, we arrive at a value similar to our 7.8 MJ per package.

Our bottom-up estimate of package delivery truck energy efficiency is fairly consistent with UPS’ reporting of 8.32 packages delivered per gallon of fuel, because UPS’ estimate includes fuel consumption by their feeder network (which we did not estimate here), and their feeder network “connects our distribution hubs to each other and to high-volume customers, our own delivery network with the familiar brown delivery vehicles, as well as third-party trucking and rail partners.” They report that the feeder network accounts for “more than half of our fuel consumption in our U.S. Domestic Package business segment”^39^. Adjusting our bottom-up estimate by half gives a value similar to UPS’ 8.32 packages delivered per gallon of fuel. This gives us confidence in our baseline value and also highlights the need for sensitivity analyses across these parameters.

Besides current Class 4 parcel delivery trucks using a range of fuels, we also estimate impacts from both vans and automobiles. Last mile delivery in an urban setting could also be accomplished with smaller, more efficient delivery vans of various sizes. To bound the vehicle performance we use a smaller van, the Nissan NV200, which has a city fuel economy of 10.2 km/l (24 mpg)^41^. A small electric cargo van is currently not available in the United States, but we test this case using a van with a 30 kWh battery achieving 100 MPGGE. A recent news article mentioned that 150 parcels were loaded onto Amazon.com urban delivery vans^42^, nearly matching our assumption of 151 packages per day for traditional parcel trucks. If delivery vans can maintain the same number of stops per mile as Class 4 trucks, then their energy consumption is 3.1 MJ per package or 37.8 packages per gallon.

To test a scenario where customers use light-duty passenger vehicles to pick-up a package from a retail store, we use the city fuel economy of exemplary gasoline (31 MPG Nissan Versa) and electric (124 MPGGE Nissan Leaf) light-duty vehicles from U.S. fuel economy information^41^. The U.S. Department of Transportation estimated that the average vehicle trip length for shopping purposes was 6.4 miles, according to the most recent survey data^43^. We use twice this length (12.8 miles or 20.6 km) as the base case roundtrip travel distance for personal package pick-up from a retail store. If the roundtrip energy is allocated to one package, the base case energy use is 50.5 MJ for the gasoline personal car and 3.7 kWh for the EV personal car. We test the sensitivity to these results by using minimum and maximum roundtrip distances for retail of 2 and 20 miles, respectively, as done in Weber et al.^27^. This sensitivity range would also likely include the uncertainty regarding allocation of per package energy and chained trips with other errands. At a roundtrip distance of 2 miles, a personal gasoline car would still have more GHGs per package than small drones in most of the U.S., but would have lower GHGs than large drones.

### Emissions from fuels

The life-cycle GHG intensities of diesel fuel, gasoline and natural gas are adapted from Argonne National Laboratory’s GREET model^23^ and Tong et al.^40^, using IPCC AR5 100-year Global Warming Potential values^44^ of 1 (CO_2_), 36 (CH_4_), and 298 (N_2_O), and are summarized in Supplementary Table 5. These include both direct values and upstream GHGs associated with extraction, transportation, and processing of fuels.

### Emissions from electricity

We use our calculated range of 3.5 km for the quadcopter and 4.2 km for the octocopter, and estimate the GHGs per trip for these distances. Our estimate of direct average energy consumption for a quadcopter with a 1 kg Li-Polymer battery is 32 J/m (0.0089 Wh/m) and our estimate for an octocopter with a 10 kg Li-Polymer battery is 266 J/m (0.0739 Wh/m).

Because of efficiency losses in the drone battery and charger, as well as in the electric power transmission and distribution systems, more than 1 unit of electricity generation is required at the power plant to provide 1 unit of usable electricity from the drone battery. We assume battery and battery charging efficiencies are each 90%, for a combined efficiency of 81%^45,46^. We use a value of 5% for transmission and distribution losses from the EPA^22^. Hence, electricity generated at the power plant, traveling the transmission and distribution system, converted at the drone charger, and traveling into and out of the battery to power the motor has an efficiency of about 77%. Our estimates reflect these efficiencies and hence electricity values are power plant-to-drone estimates. The emissions from drone electricity are the product of electricity per meter (including charging and distribution losses), trip distance, and subregional non-baseload electricity emissions factors from the e-GRID dataset from the U.S. Environmental Protection Agency.

There are a variety of methods to allocate GHG and air pollutant emissions to local electricity use, and each has benefits and drawbacks^20,21,47–51^. The regional yet interconnected architecture of the electricity system, coupled with hourly, seasonal, inter-annual variation, and long-term change in fuel use and asset composition introduces temporal and spatial uncertainty^20,21,52^. New demand from drones would be charged by electricity generating units at the margin, rather than baseload electricity generating units. Because the GHG emissions intensity of electricity varies regionally, and for the desire to have open and replicable data inputs, we use EPA’s e-GRID annual direct non-baseload GHG emissions^22^ to present the range of emissions in each North American Reliability Corporation (NERC) region (shown in Supplementary Table 6), in each e-GRID subregion (shown in Supplementary Table 7), and for the United States average. When comparing the results, e-GRID subregions have the advantage of smaller geographic boundaries, whereas NERC regions reduce some of the uncertainty from electricity trading across a larger area. To represent the range, we estimate the emissions using both NERC regions and e-GRID subregions. For the main figures, we report the range from California (e-GRID subregion WECC California (CAMX) to the highest value in Missouri (e-GRID subregion SERC Midwest (SRMW). We note that e-GRID subregion WECC Northwest (NWPP) has about 3% less GHGs than California (resulting in similar outcomes). For completeness, we report drone GHG results for each NERC region and e-GRID subregion in Supplementary Figs. 10–13. The values are consistent with the endpoints selected for presentation in Fig. 5. As done with other emissions above, methane and nitrous oxide emissions are converted to CO_2_-equivalent with a 100-year global warming potentials (GWP) from IPCC AR5^44^. A map of the NERC regions is shown in Supplementary Fig. 5 and a map of EPA e-GRID subregions is shown in Supplementary Fig 6. Because the fuel mix and emissions of the electricity grid is likely to evolve toward lower emissions over time, and because reduced relative natural gas prices can result in reduced electricity emissions as gas substitutes for coal in the near-term^53^, we include a low-carbon case using 200 g/kWh. We also present the spectrum of results as GHG intensity of electricity varies from 100 to 1000 g/kWh in the sensitivity analysis. In Supplementary Fig. 9, we show average emissions factors for electricity from several selected continents and countries.

Emissions and other environmental impacts are also generated in the manufacturing, construction and decommissioning of specific electricity and fuels infrastructure, as well as in the extraction, processing and transportation of energy^54–61^. Hence upstream emissions from electricity production vary both within and across regions based on time of day, season, fuel mix, methane leakage rates, and technological change. Argonne National Laboratory’s GREET model estimates upstream emissions from average U.S. power plants by fuel type (see Supplementary Table 8). We estimate the weighted percentage of non-baseload generation by fuel in each NERC region and e-GRID subregion per the e-GRID guidance, and multiply these percentages by the upstream emissions values for each fuel from Argonne’s GREET model. This yields an upstream value in g GHG/kWh for non-baseload generation in each NERC region and e-GRID subregion. Values for the weighted percentage of non-baseload generation by fuel in each NERC region are shown in Supplementary Table 9 and in e-GRID subregions in Supplementary Table 10. Direct, upstream, and life-cycle emissions in each NERC region are shown in Supplementary Table 11, and in each e-GRID subregion in Supplementary Table 12. The uncertainties involved in estimating upstream emissions from non-baseload electricity in regional electricity grids are compounded across assumptions about resource extraction, transport, and power plant type. We tested the sensitivity of the results to the distributions of upstream GHGs from fossil fuel-fired power plants used in Tong et al.^40^ and Abrahams et al.^62^, which did not measurably change the results.

### Emissions from battery production

The impacts of manufacturing and raw material extraction for lithium-ion batteries can be an important portion of the life-cycle impacts of electrified transportation. These impacts depend on the battery chemistries, manufacturing and recycling processes, useful life, and other parameters^24,63–68^. For this analysis, we assume Li–Po batteries will have similar impacts to lithium-ion phosphate batteries. We use an estimate from Argonne’s GREET model^23^ for the embodied energy and GHG emissions for 1 kg of lithium-ion phosphate batteries of 75 MJ and 4.7 kg of GHG, respectfully. We assume these impacts are distributed equally per drone trip over the cycle life of the battery. Testing a range of cycle life assumptions did not measurably affect the results from the small drone, but battery emissions from large drones would be affected by either better or worse than expected cycle life. We also use an estimate from GREET for 1 kg of lithium cobalt oxide batteries of 154 MJ and 10.4 kg of GHG. For ground vehicles, battery emissions are allocated per km and then per package. If early battery replacement is required, emissions would increase.

### Emissions from warehouses

UPS reports a total global electricity consumption of about 1628 GWhs, 4365 TJs of natural gas, and 4.7 billion packages delivered in 2015^39^. Using these numbers, the UPS metrics for 2015 are 0.35 kWh per package for electricity and 0.93 MJ per package of natural gas. Given the large uncertainty involved in accounting boundaries, local climate, fuels, and operations, we use these UPS metrics as a base case and test this assumption in the sensitivity analysis. Emissions from warehousing are the product of these metrics and regional electricity emissions factors or life-cycle emissions from stationary combustion of natural gas. UPS reports 1970 g GHG per U.S. domestic package, resulting from all stationary and mobile combustion in their supply chain, and they note that more than half of these emissions are from air operations. Our bottom-up estimate of about 880 g GHG for the local diesel truck delivery (excluding upstream fuel production) and warehousing portion is within the values expected, given the reporting by UPS.

The energy use and environmental impacts of drone delivery depend substantially on the drones’ range and manner of implementation. As explained in the main text, the degree of extra warehousing required for drone delivery dominates the comparison of drone- and truck-based scenarios. For servicing an urban area with on-demand delivery, two main approaches have been proposed. The first is to locate distribution centers such that all of the service area is within delivery range of a distribution center. The second is to establish waystations such that drones can fly from one to another and exchange batteries or trade packages in a series of hops from distribution center to destination. In the case of connecting one way station to another, the effective range of the drone is reduced somewhat because it flies the whole trip with a package instead of only the outbound trip. However, this difference is small, and we neglect it.

To give a sense of the number of nodes required for a typical urban area, Fig. 3 shows a hypothetical coverage map for the city of San Francisco and for the populated areas of the greater San Francisco Bay Area. The service area is defined by a circle around each node (warehouse or way station) with radius equal to the range of the drone, where range is defined as the farthest distance from the warehouse to which a drone can deliver a package and still return to the warehouse. The nodes were placed manually based on a drone range of 3.5 km and covering most significant regions with population density above the lowest category (>0.5 people per 30×30 m square), yielding rough estimates only. We find that about four nodes can service the small, dense city of San Francisco, but about 112 nodes are needed to service the Bay Area.

More generally, the problem of covering a defined area with the minimum number of equally sized circles has been explored for a variety of applications, including placement of fire sensors and cellular telephone towers. The solution depends on the boundary shape and relative size of the circles and boundary. Square and hexagonal packings are each optimal in certain circumstances, however hexagonal packing tends to be best over larger areas^69^. Li et al. provide a formula to calculate the minimum number of nodes required to cover a square area with hexagonal packing. The results for an area 16 km on a side (representing a medium-sized city) and varying drone range are shown in Supplementary Fig. 7. We see that the number of required nodes drops quickly with improvements in range from current technology. For example, doubling the range from 3.5 to 7 km decreases the number of nodes by a factor of 3.5. This illustrates the strong economic and environmental incentive to increase the range of the drones, if they are to be deployed from local warehouses. In our base case, we assume drone and van pathways require twice as much warehouse space (and energy) than conventional truck pathways. For our best case, we assume no difference in warehouse energy, and for our worst case, we assume drones and vans will need four times as much warehouse energy.

Overall, we find that a metropolitan area currently served by one regional distribution center could easily require dozens of new local warehouses to achieve the on-demand delivery model proposed for drones. In our base case scenario, we assume that the local warehouses constitute a new and additional stage in the logistics system and that the total storage space in local warehouses is comparable to the space in the distribution center that serves them. Conceivably, a retailer selling a high volume of a small number of products could bypass the regional distribution center and stock local warehouses directly from suppliers. This would reduce the impact of the new local warehouses. On the other hand, a retailer stocking a large number of distinct products might have to replicate its inventory many times to make the products available at each local warehouse.

### Data availability

The datasets and code generated in the current study are available from the corresponding authors on reasonable request.

## Electronic supplementary material

Supplementary Information
