# Author Correction: Energy use and life cycle greenhouse gas emissions of drones for commercial package delivery

**DOI:** 10.1038/s41467-018-03457-9

**Published:** 2018-03-08

**Authors:** Joshuah K. Stolaroff, Constantine Samaras, Emma R. O’Neill, Alia Lubers, Alexandra S. Mitchell, Daniel Ceperley

**Affiliations:** 1 E Program, Global Security, Lawrence Livermore National Laboratory, Livermore, CA 94551 USA; 2 Civil & Environmental Engineering, Carnegie Mellon University, Pittsburgh, PA 15213 USA; 3 Advanced Technology and Systems Division, SRI International, Menlo Park, CA 94025 USA; 4 Chemical and Biological Engineering, University of Colorado at Boulder, Boulder, CO 80309 USA; 5 Present Address: LeoLabs, Inc., PO Box 998, Menlo Park, CA 94026 USA

Correction to: *Nature Communications* (2018) 10.1038/s41467-017-02411-5, published online 13 February 2018

In the original version of this Article, the first sentence of the sixth paragraph of the “Comparing emissions” section, the Results originally incorrectly read as ‘In the base case, delivery of a small (0.5 kg) package with the small quadrotor drone has lower impacts than delivery by diesel truck, ranging from a 59% reduction in GHGs in California, to a 17% reduction in Missouri’. The correct version states ‘54%’ instead of ‘59%’ and ‘23%’ instead of ‘17%’.

The fourth sentence of the same paragraph originally incorrectly read as ‘In the base case, delivery of a medium-sized (8 kg) package has 17% lower GHGs than delivery by truck in California, is about equivalent to delivery trucks for the U.S. average electricity mix, but has 77% higher GHGs than truck delivery in Missouri, which has a carbon-intensive electricity grid’. The correct version states ‘In the base case, delivery of a medium-sized (8 kg) package has 9% lower GHGs than delivery by truck in California, is about 24% higher than delivery trucks for the U.S. average electricity mix, and has 50% higher GHGs than truck delivery in Missouri, which has a carbon-intensive electricity grid.

The last sentence of the seventh paragraph of the same section originally incorrectly read as ‘Because of the importance of electricity used to power the octocopter, charging with low-carbon electricity of 200 g GHG/kWh can reduce delivered package GHGs by 34% compared to diesel trucks’. The correct version states ‘37%’ instead of ‘34%’.

These errors have been corrected in both the PDF and HTML versions of the Article.

