# Supplementary material for: Energy use and life cycle greenhouse gas emissions of drones for commercial package delivery
Source: Nat Commun. 2018 Feb 13;9:409. doi: 10.1038/s41467-017-02411-5 (PMC5811440; doi:10.1038/s41467-017-02411-5)
Supplement: Supplementary file 1 — Supplementary Information [file 41467_2017_2411_MOESM1_ESM.pdf]

Supplementary Information

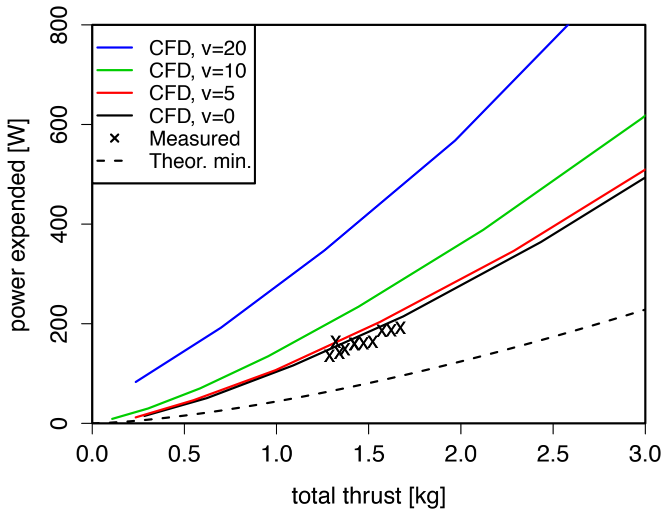

Supplementary Figure 1: Power vs. thrust relationship for the test model quadcopter. "CFD" lines are based on results provided by the rotor manufacturer based on a Computational Fluid Dynamics model. The power-thrust relationship changes depending on the incident air velocity, *v.* Results for several velocities are shown. Measured data have an average implied power efficiency of 53% compared to the theoretical minimum (Eq. 1). measured tilt of the drone, assuming force equilibrium (steady flight). Dashed line is the best-fit regression line, showing the drag coefficient decreases with speed.

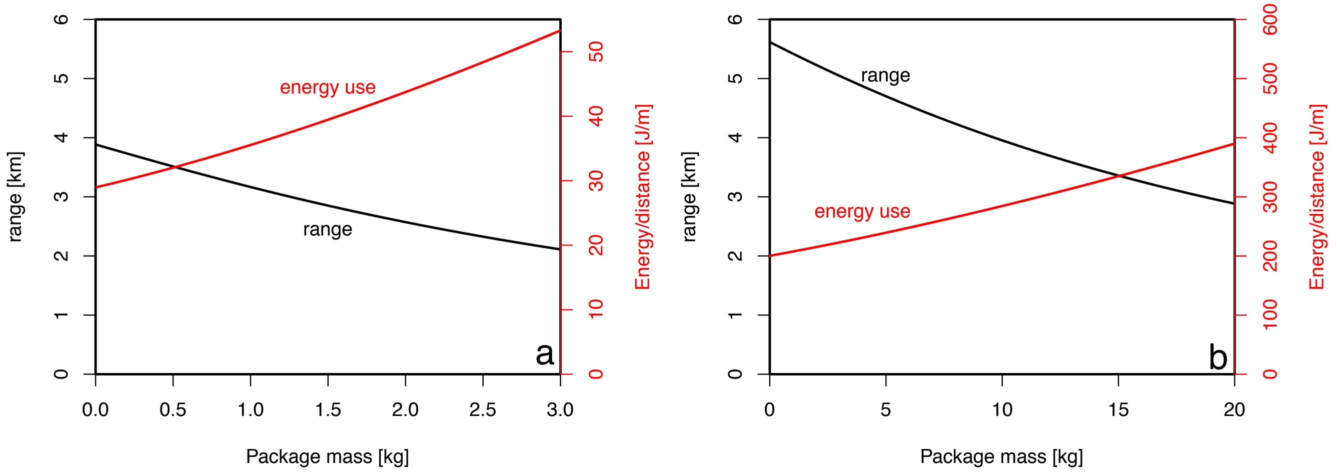

Supplementary Figure 2: Range and energy use as a function of package mass for the quadcopter (a) and octocopter (b). The maximum design payload masses for the quadcopter and octocopter test models are 0.5 and 8 kg, respectively. Drones can be designed for larger payloads, but would require more powerful motors, larger voltages, etc.

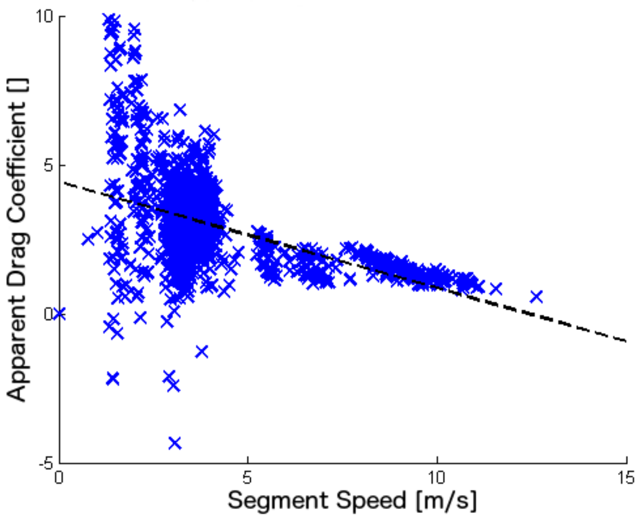

Supplementary Figure 3: Implied empirical drag coefficient vs. drone speed. Each point represents the average for a flight segment. The drag coefficient is based on the onboard measured tilt of the drone, assuming force equilibrium (steady flight). Dashed line is the best-fit regression line, showing the drag coefficient decreases with speed.

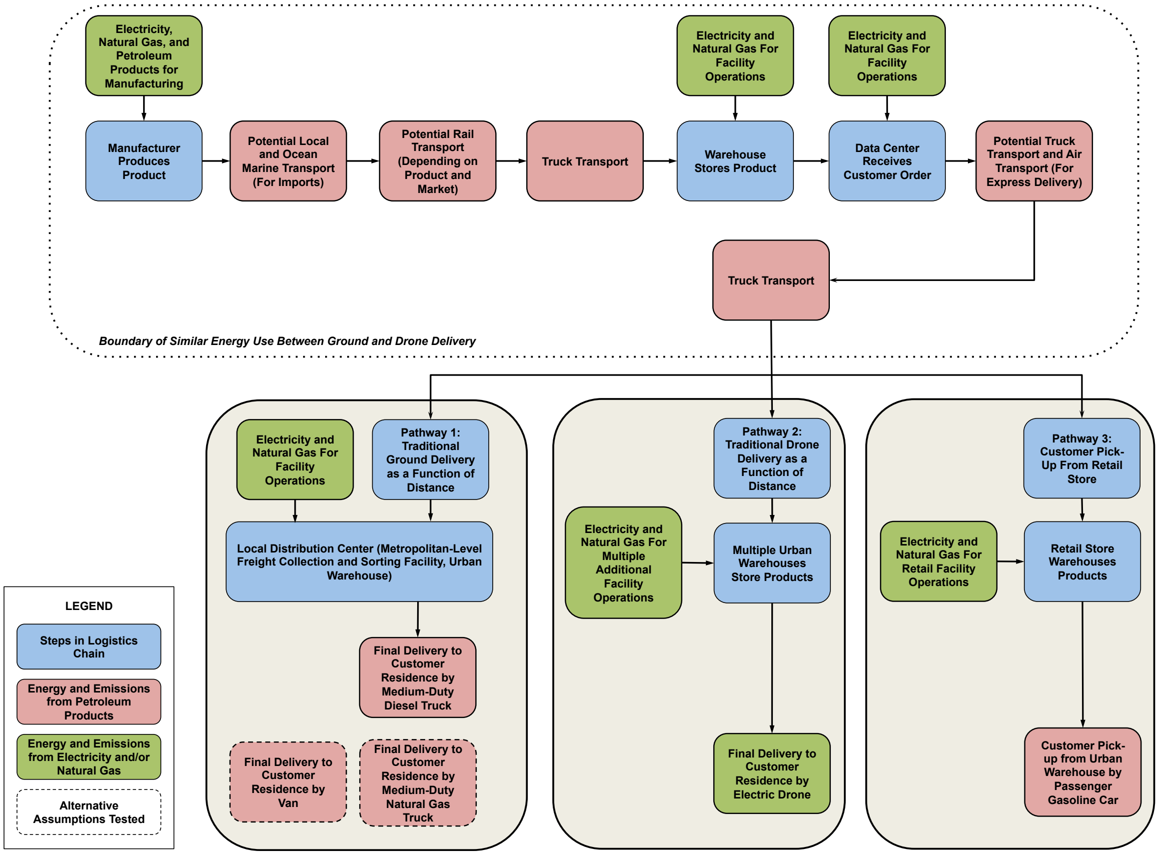

Supplementary Figure 4: System Boundary for Estimating Energy and Emissions Across Three Local Delivery Scenarios. GHG emissions are estimated across three broad delivery pathways. These include final delivery by medium-duty delivery truck, representing the most common existing logistics pathway; Package storage in additional urban warehouses, with final delivery by electric-powered drone; and customer pick-up from retail store or urban warehouse by gasoline or electric passenger vehicle

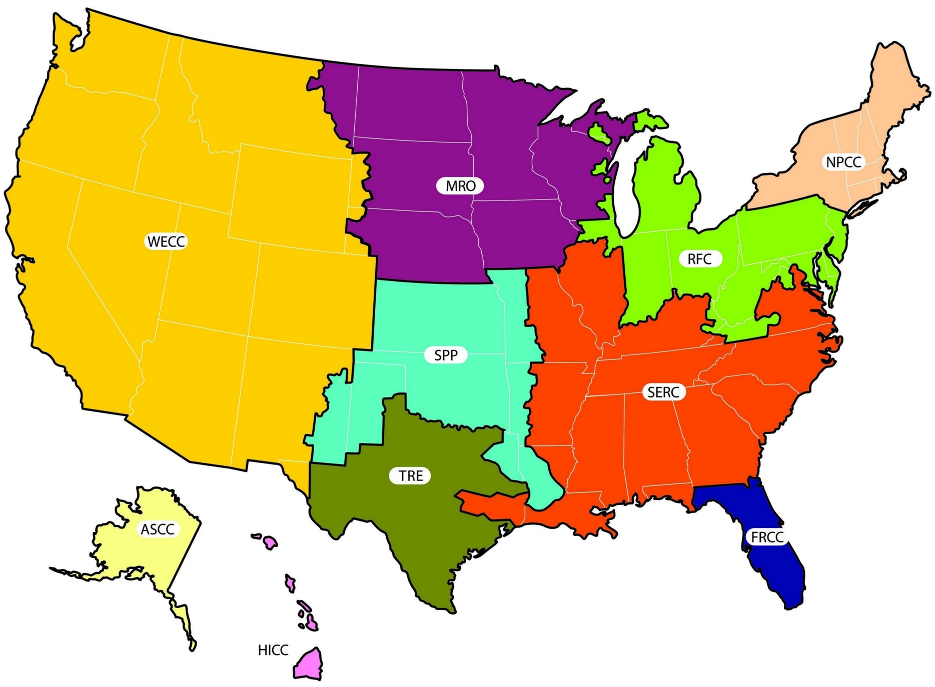

Supplementary Figure 5: NERC Region Map used by EPA eGRID[1]

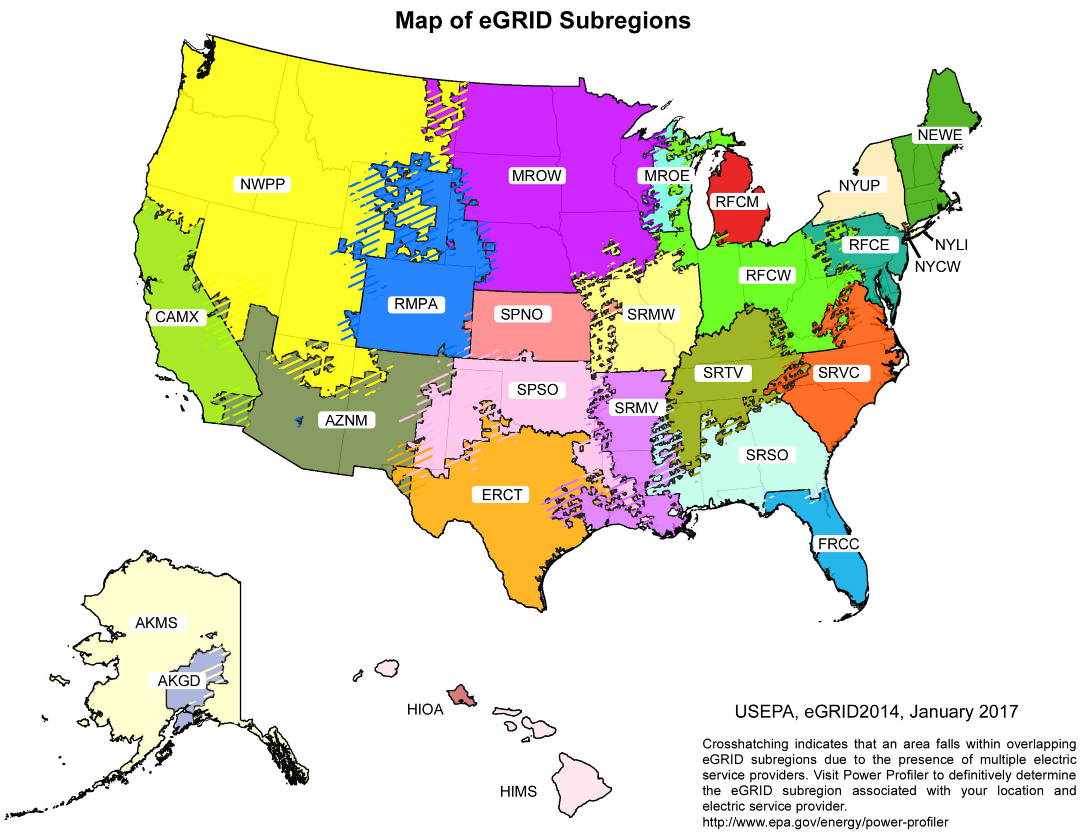

Supplementary Figure 6: EPA Subregion Map used by EPA eGRID[1]

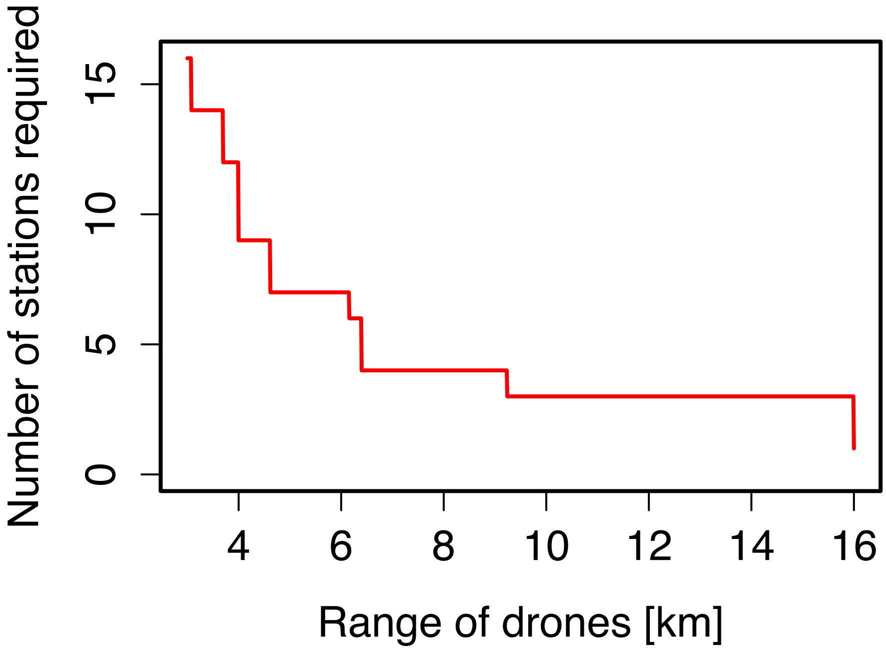

Supplementary Figure 7: Minimum number of warehouses or way stations required to service a square area 16 km on a side. Stations are arranged in a hexagonal pattern.

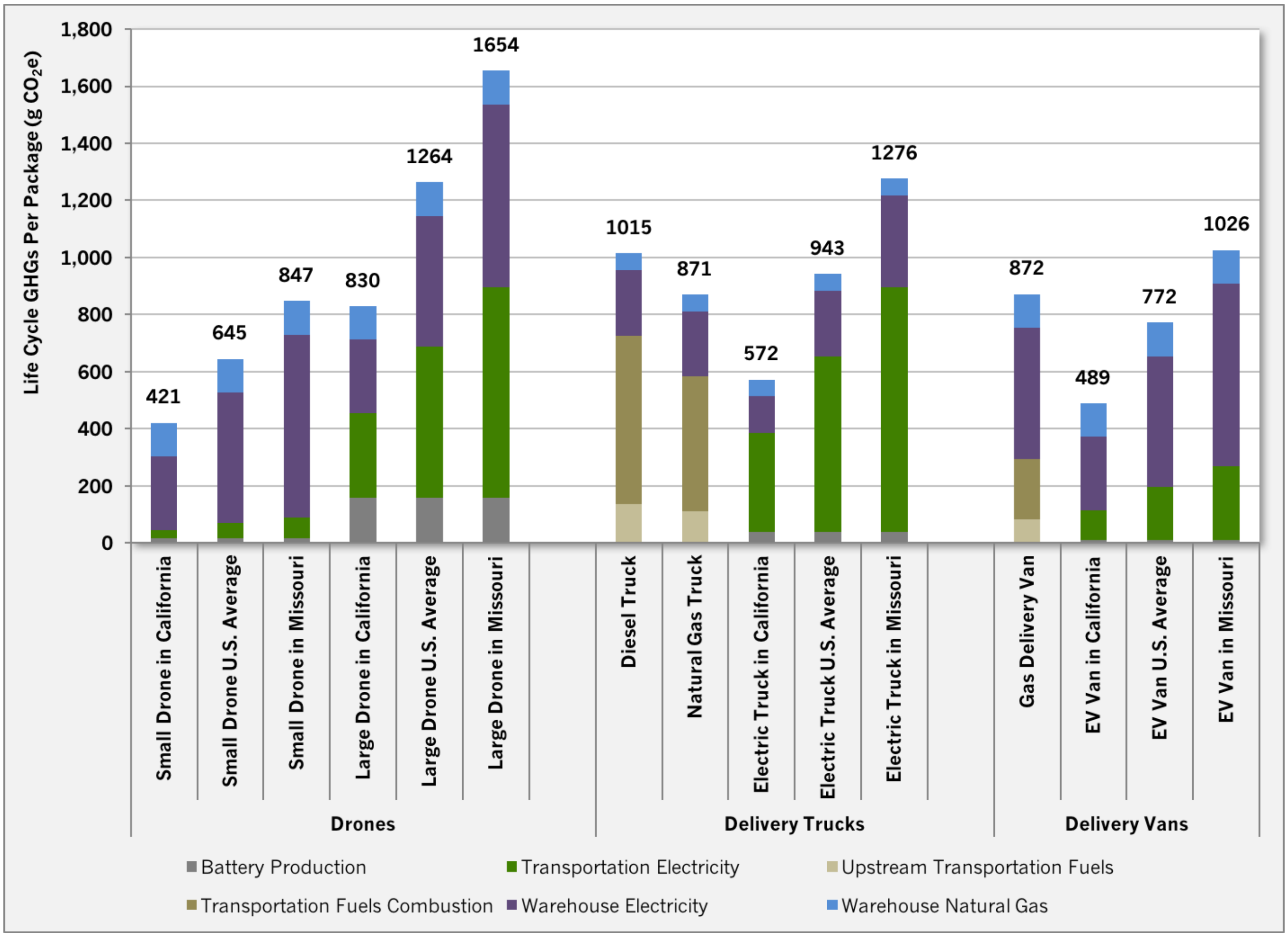

Supplementary Figure 8: Life cycle GHGs per package across drone, truck, and van pathways for diesel, natural gas, and electric vehicles (EVs). Total g CO_2-eq per package values shown above each bar.

.

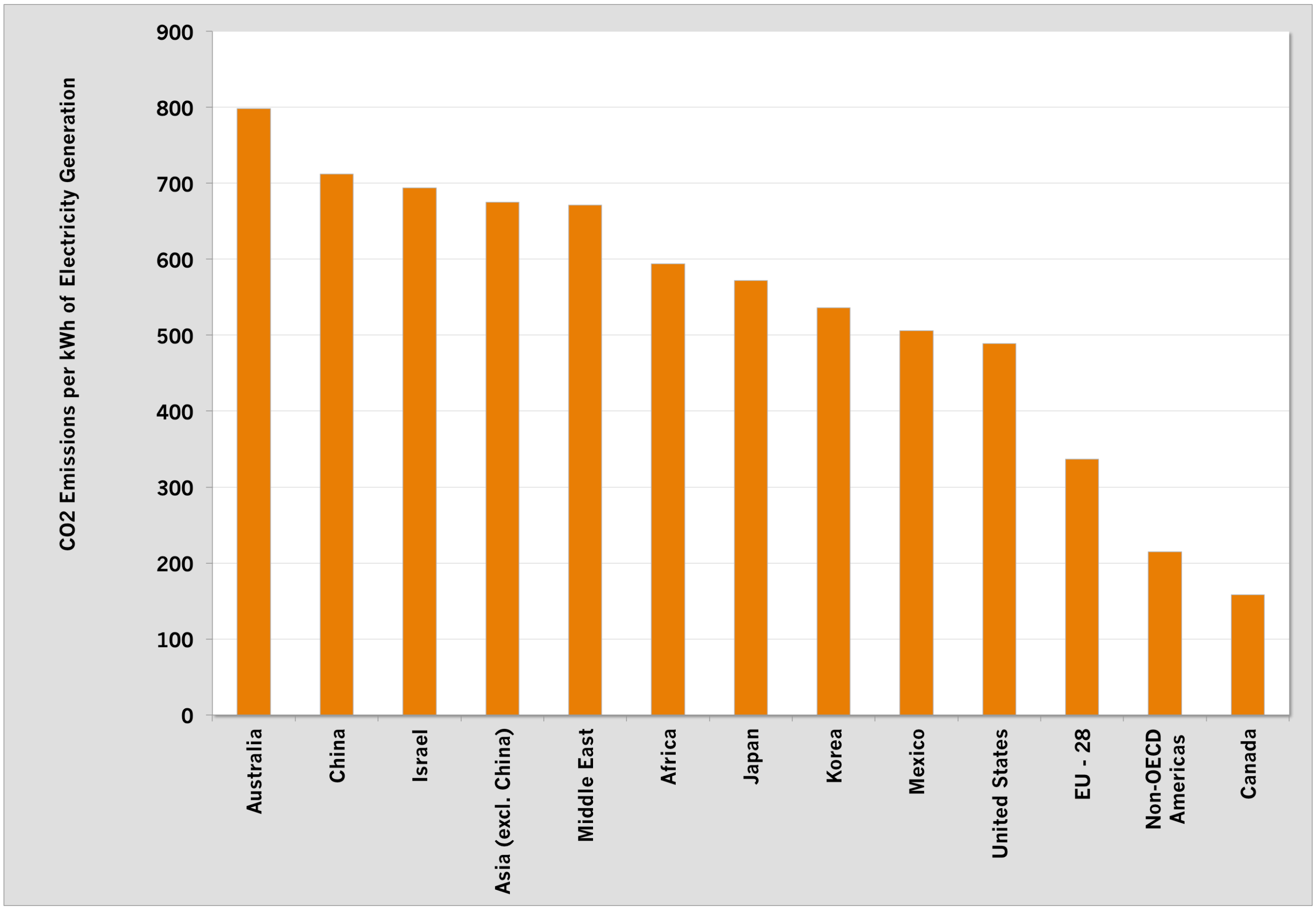

Supplementary Figure 9: CO_2 emissions per kWh of electricity for selected continents and countries. Data from IEA [2].

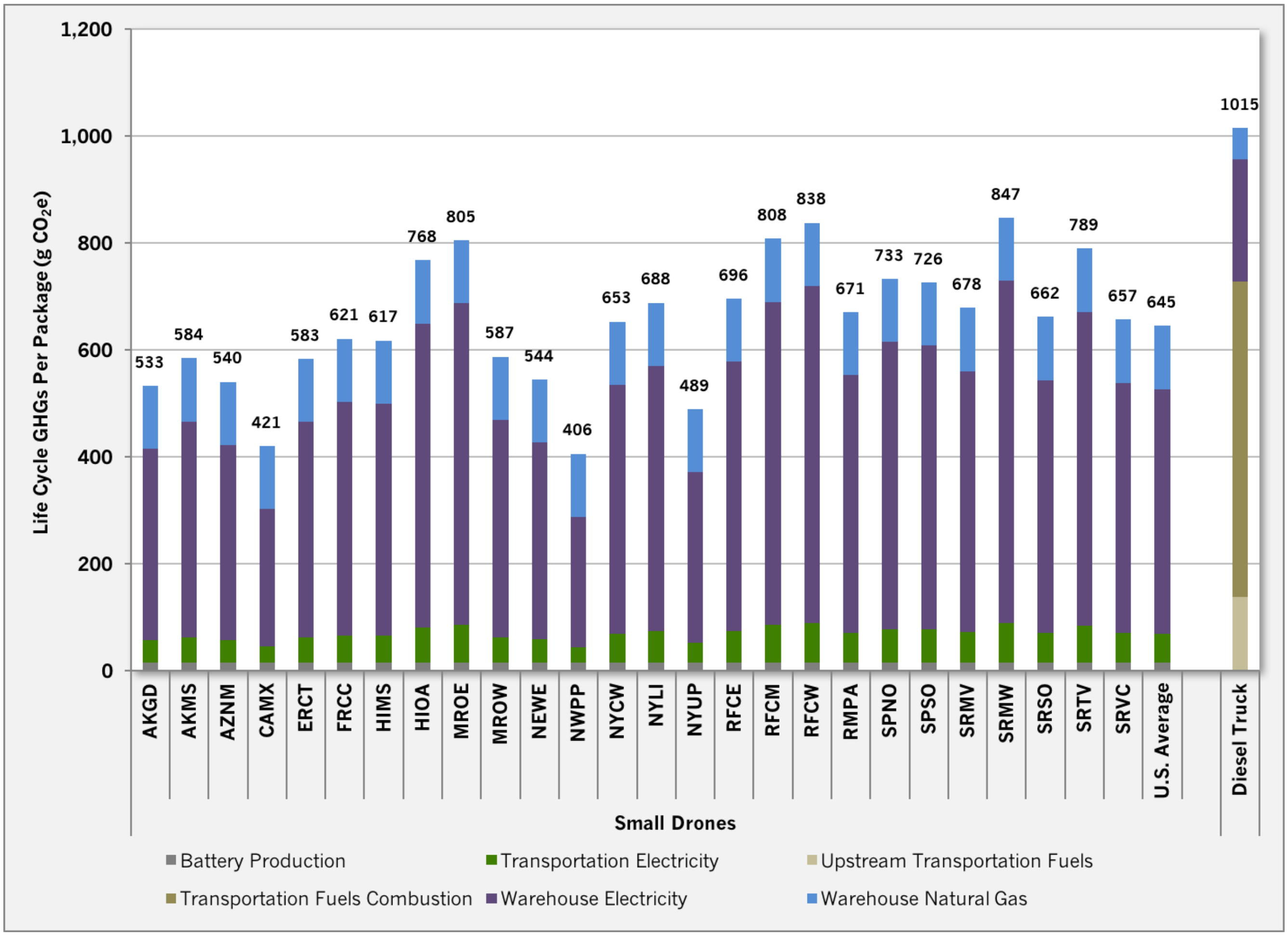

Supplementary Figure 10: Life cycle GHGs per package for small drones across e-GRID subregions, compared to diesel trucks. Total g CO_2-eq per package values shown above each bar.

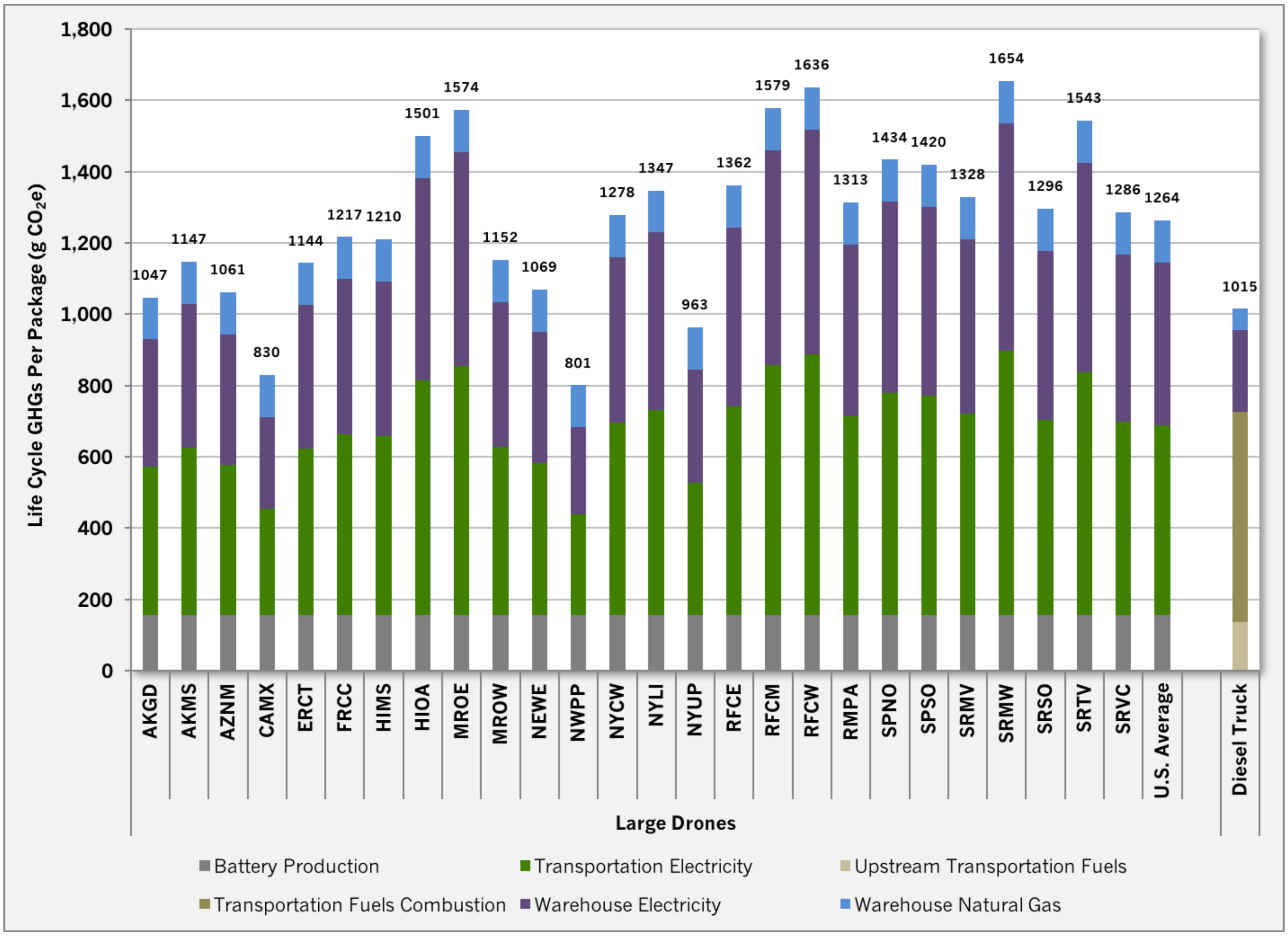

Supplementary Figure 11: Life cycle GHGs per package for large drones across e-GRID subregions, compared to diesel trucks. Total g CO_2-eq per package values shown above each bar.

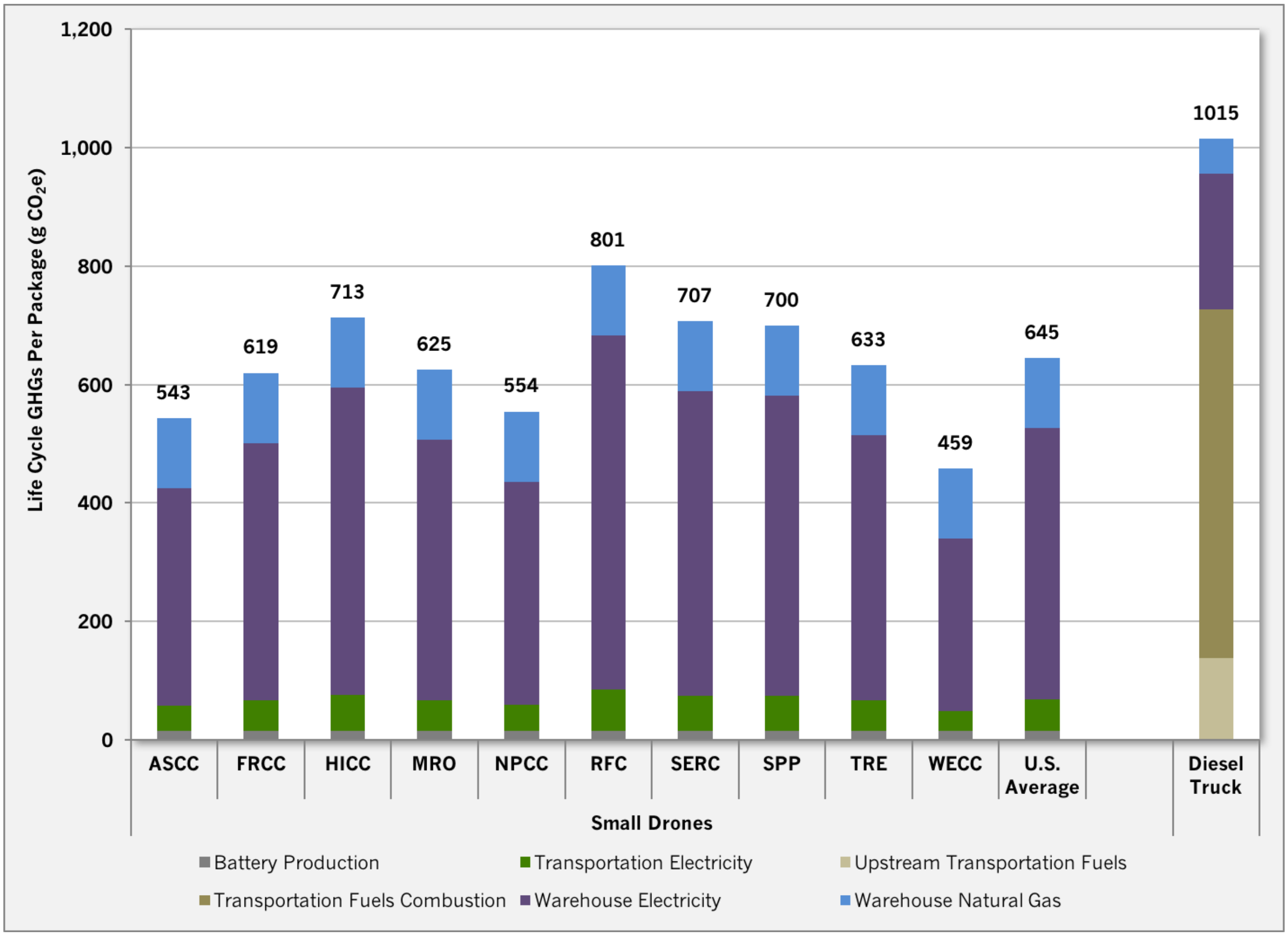

Supplementary Figure 12: Life cycle GHGs per package for small drones across NERC regions, compared to diesel trucks. Total g CO_2-eq per package values shown above each bar.

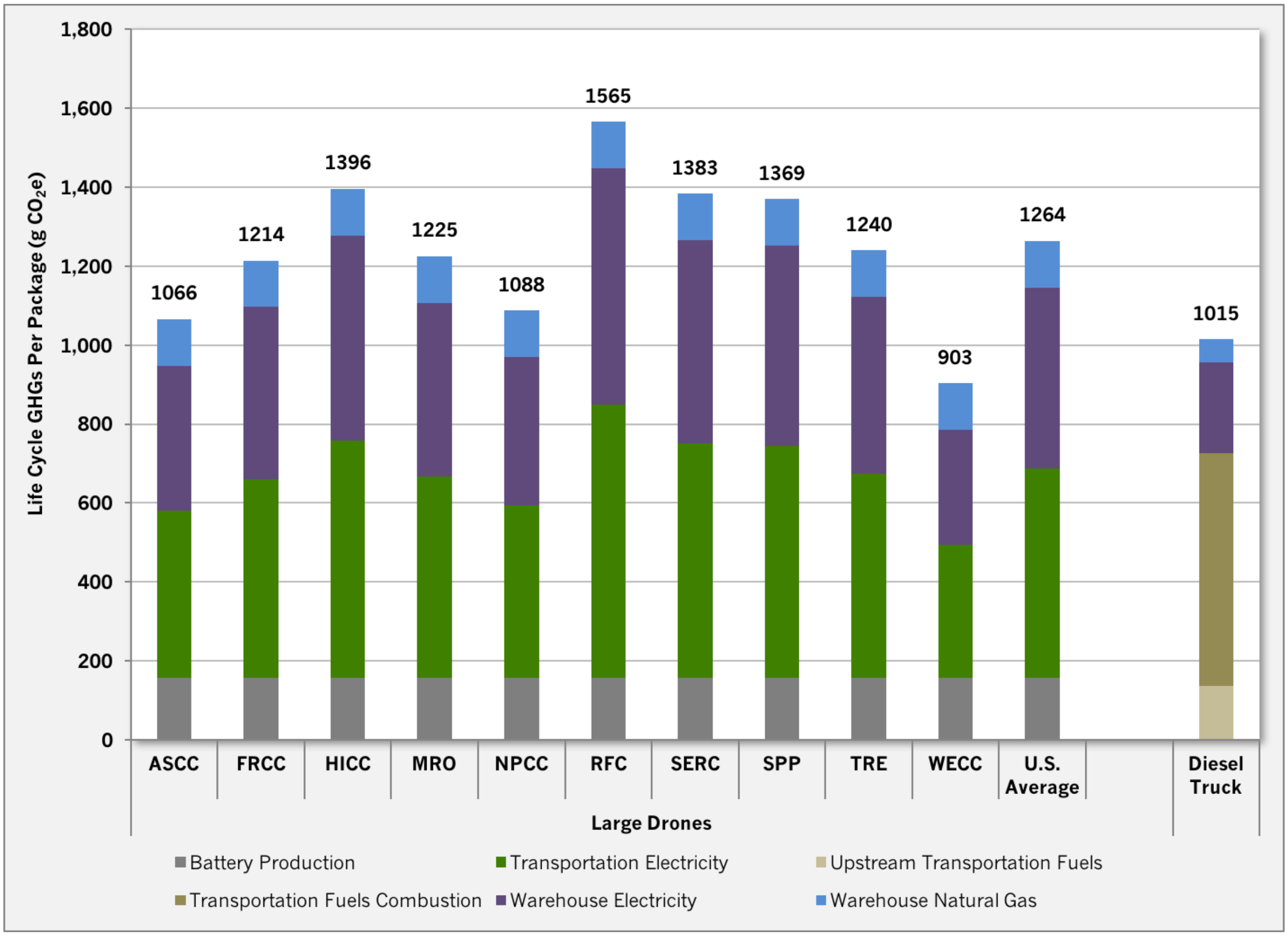

Supplementary Figure 13: Life cycle GHGs per package for large drones across NERC regions, compared to diesel trucks. Total g CO_2-eq per package values shown above each bar.

Supplementary Table 1: Base case parameter values used in the drone energy use model

	Quadcopter	Octocopter
mass of copter body [kg]	1.07	7
mass of payload [kg]	0.5	7
mass of battery [kg]	1	10
number of rotors	4	8
rotor diameter [m]	0.254	0.432
projected area of drone body [m^2]	0.0599	0.224
projected area of additional battery [m^2]	0.0037	0.015
projected area of package [m^2]	0.0135	0.0929
drag coefficient of body	1.49	1.49
drag coefficient of batteries	1	1
drag coefficient of package	2.2	2.2
overall power efficiency	0.7	0.7
energy density of battery [J/kg]	540,000	540,000
energy density of future battery technology [J/kg]	900,000	900,000
mass density of battery [kg/m^3]	3,300	3,300
fraction of battery energy spent on a mission	0.5	0.5
battery safety factor (total capacity / nominal requirement)	1.2	1.2
base case velocity [m/s]	10	10

Supplementary Table 2: Summary of current and proposed energy storage technologies.

Battery Chemistry/Fuel Cell / Combustion Fuel	Theoretical Maximum Energy Density (Wh per kg / Wh per L)	Practical Energy Density (Wh per kg /Wh per L)	Proposed Maximum # of cycles	Depth of Discharge	Represent-ative Range [km]
Metal Battery Technology					
Nickel Metal Hydride	800 / 1,940 [3]	80 [4] / 190[a]	200,000 [3]	80% [5]	1.8
Nickel Zinc	370 / 740 [3]	100 [6] / 200[a]	250 [5]	60% [5]	2.3
Zinc-Air	700 [7] / NA	400 [7]/ NA	200 [5]	80% [5]	9.1
Lithium-Air	5,000 / 1,000 [8]	1,000 [8]/ 200[a]	1 [8]	40% [9]	23
Lithium-Sulfur	2,600 [10] / 2,800 [11]	NA / NA	400 [8710]	NA	NA
Li-ion Battery Technology					
Lithium Polymer	890 / 1440 [5]	107 / 170 [12]	300 [5]	80% [5]	3.5
Lithium Cobalt (LCO)/Carbon	990 / 4,980 [13]	220 [14]/ 1,090[a]	1200 [5]	100% [5]	5.0
Lithium Manganese (LMO)/Carbon	460 / 1,890 [13]	160 [15]/ 660[a]	300 [5]	80% [5]	3.6
Lithium Iron Phosphate (LFP)/Carbon	580 / 2,080 [13]	130 / 250 [16]	3,000 [16]	100% [16]	3.0
Fuel Cell Technology Fuels					
Hydrogen (liquid)	33,300 / 2,360 [17]	16,650 [18] / 1,180[a]	9,000[b]	100%[c]	--
Hydrogen (200 bar)	33,300 / 530 [17]	16,650 [18] / 270[a]	9,000[b]	100%[c]	11
Methanol	5,550 / 4,390 [17]	2,220 [18] / 1,760[a]	9,000[d]	100%[c]	20
Hydrocarbon Combustion Fuels					
Gasoline	12,400 / 9,100 [18]	4,710[e] / 3,450[e]	80,470[f]	100%[c]	--
Methanol	5,550 / 4,390 [17]	2,110[e] / 1,660[e]	80,470[f]	100%[c]	--
Nitromethane	3,230 [19] / 3,670[g]	1,220[e] / 1,390[e]	80,470[f]	100%[c]	--
Synthetic Oil	2,820 [20] / 2,490[g]	1,070[e] / 940[e]	80,470[f]	100%[c]	--
Glow Fuel[h]	4,310[i] / 3,930[i]	1,640[e] / 1,500[e]	80,470[f]	100%[c]	--

[a] calculated from density determined from theoretical values
[b] calculated from 2,500 hour maximum lifetime[21], with a cycle consisting of 16.6 min
[c] considering full use of a fuel tank
[d] calculated from 3,000 hour maximum lifetime[22], with a cycle consisting of 16.6 min
[e] calculated from efficiency of internal combustion engine[23]
[f] calculated from 250,000 mile maximum lifetime, with a cycle consisting of 5 km
[g] calculated from density of nitromethane[19] and synthetic oil (type 15W-40)[24]
[h] composed of 50% methanol, 30% nitromethane, and 20% synthetic oil
[i] calculated from mixture characteristics

Supplementary Table 3: Parameters for the calculation of effective energy density of hydrogen and methanol fuel cell systems for use with drones.

Parameter	Hydrogen Fuel Cell	Methanol Fuel Cell
Energy capacity basis [W•h]	500	500
Fuel mass [g]	15	90
Fuel volume [L]	0.9*	0.1
Fuel tank mass [g]	750 [25]	210 [26]
Fuel cell mass [g]	275 [27]	275 [27]
System energy density [W•h/kg]	480	867

* Assuming hydrogen pressure of 200 bar.

Supplementary Table 4: Vehicle and Fuels Parameters. Drone values estimated in this study. Other vehicle and fuels parameters adapted from [28–30]. Estimated MJ/package values shown reflect base case assumptions in this analysis.

Gallon of gasoline	118.4	MJ
Gallon of diesel	135.5	MJ
Class 4 Diesel package delivery truck	11.5	MPGDE
Class 4 Diesel package delivery truck	7.32	MJ/km
Class 4 Diesel package delivery truck	7.8	MJ/package
Class 4 CNG package delivery truck	10.8	MPGDE
Class 4 CNG package delivery truck	7.8	MJ/km
Class 4 CNG package delivery truck	8.3	MJ/package
Class 4 EV package delivery truck	34.5	MPGDE
Class 4 EV package delivery truck	3.17	MJ/km
Class 4 EV package delivery truck	3.44	MJ/package
Gasoline light-duty delivery van	24	MPG
Gasoline light-duty delivery van	3.06	MJ/km
Gasoline light-duty delivery van	3.3	MJ/package
Gasoline light-duty personal car	31	MPG
Gasoline light-duty personal car	2.45	MJ/km
Gasoline light-duty personal car	50.5	MJ/package
Electric light-duty delivery van	100	MPGGE
Electric light-duty delivery van	0.96	MJ/km
Electric light-duty delivery van	1.02	MJ/package
EV light-duty personal car	124	MPGGE
EV light-duty personal car	0.77	MJ/km
EV light-duty personal car	15.9	MJ/package
Small Drone	0.04	MJ/km
Small Drone	0.29	MJ/package
Large Drone	0.34	MJ/km
Large Drone	2.9	MJ/package

Note: electric vehicle energy use includes losses in transmission, distribution, and charging, as discussed.

Supplementary Table 5: Emissions Factors for Fuels. Data are adapted from [30] and [28].

	Combustion GHGs [g CO_2-eq/MJ]	Upstream GHGs [g CO_2-eq/MJ]	Total GHGs [g CO_2-eq/MJ]
Diesel fuel	75	18	93
Gasoline (reformulated E10)	65	25	90
Compressed Natural Gas	57	20	77
Natural Gas for Heating	57	13	70

Supplementary Table 6: Non-Baseload Direct Emissions Factors Used for Electricity

NERC Region	Acronym	CO_2 [g/kWh]	CH_4 [g/kWh]	N_2O g/kWh]	GHGs [g CO_2e/kWh]
Alaska Systems Coordinating Council	ASCC	419.9	0.039	0.006	423
Florida Reliability Coordinating Council	FRCC	530.6	0.074	0.010	531
Hawaiian Islands Coordinating Council	HICC	593.6	0.118	0.019	594
Midwest Reliability Organization	MRO	550.5	0.128	0.019	551
Northeast Power Coordinating Council	NPCC	442.9	0.065	0.009	443
Reliability First Corporation	RFC	772.8	0.153	0.022	773
SERC Reliability Corporation	SERC	648.8	0.121	0.017	649
Southwest Power Pool	SPP	639.0	0.112	0.016	639
Texas Regional Entity	TRE	551.1	0.066	0.009	551
Western Electricity Coordinating Council	WECC	326.8	0.048	0.007	327
U.S. Non-Baseload	U.S.	568.4	0.099	0.014	568
Future Low-Carbon Grid Scenario					200

Note: Data for Non-Baseload Emissions factors for NERC regions and the U.S. are from US EPA[1]. The data are from the 2017 version of eGRID, which use 2014 data. CH4 and N2O global warming potentials from IPCC AR5[31] are used. Future low-carbon assumption represents sensitivity case for low-carbon regional electricity grid.

Supplementary Table 7: Alternative Non-Baseload Direct Emissions Factors for Electricity

EPA eGRID Subregion	EPA Subregion Acronym	Corresponding NERC Region Acronym	GHGs [g CO_2e/kWh]	eGrid- NERC % Difference
ASCC Alaska Grid	AKGD	ASCC	412	-2.7%
ASCC Miscellaneous	AKMS	ASCC	459	7.8%
WECC Southwest	AZNM	WECC	429	23.9%
WECC California	CAMX	WECC	272	-20.1%
ERCOT All	ERCT	TRE	485	-13.6%
FRCC All	FRCC	FRCC	532	0.3%
HICC Miscellaneous	HIMS	HICC	472	-25.7%
HICC Oahu	HIOA	HICC	662	10.4%
MRO East	MROE	MRO	780	29.4%
MRO West	MROW	MRO	503	-9.5%
NPCC New England	NEWE	NPCC	430	-2.9%
WECC Northwest	NWPP	WECC	264	-23.7%
NPCC NYC/Westchester	NYCW	NPCC	567	21.9%
NPCC Long Island	NYLI	NPCC	608	27.2%
NPCC Upstate NY	NYUP	NPCC	364	-21.8%
RFC East	RFCE	RFC	632	-22.3%
RFC Michigan	RFCM	RFC	782	1.2%
RFC West	RFCW	RFC	822	6.0%
WECC Rockies	RMPA	WECC	605	46.0%
SPP North	SPNO	SPP	689	7.3%
SPP South	SPSO	SPP	673	5.0%
SERC Mississippi Valley	SRMV	SPP	608	-5.1%
SERC Midwest	SRMW	SERC	837	22.5%
SERC South	SRSO	SERC	591	-9.8%
SERC Tennessee Valley	SRTV	SERC	759	14.5%
SERC Virginia/Carolina	SRVC	SERC	583	-11.3%
Future Low-Carbon Grid Scenario			200	--

Note: Data for Non-Baseload Emissions factors for EPA eGRID subregions are from US EPA[1]. The data are from the 2017 version of eGRID, which use 2014 data. Corresponding NERC regions shown are the best match and some small overlaps could occur in some cases. CH_4 and N_2O global warming potentials from IPCC AR5[31] are used. Future low-carbon assumption represents sensitivity case for low-carbon regional electricity grid.

Supplementary Table 8: Upstream Emissions Factors for Electricity Fuels. Values are from are from Argonne National Laboratory [30].

	Upstream Mean [g CO_2-eq/kWh]
Coal Electricity	74
Natural Gas Electricity	95
Oil-Fired Electricity	149

Supplementary Table 9: Non-Baseload Weighted Fuel Mix by NERC Region

NERC Region	Acronym	Coal	Natural Gas	Oil
Alaska Systems Coordinating Council	ASCC	12.2%	73.6%	14.2%
Florida Reliability Coordinating Council	FRCC	22.3%	76.4%	1.3%
Hawaiian Islands Coordinating Council	HICC	0.9%	0.0%	99.1%
Midwest Reliability Organization	MRO	81.8%	17.3%	0.9%
Northeast Power Coordinating Council	NPCC	12.0%	82.9%	5.1%
Reliability First Corporation	RFC	70.7%	27.7%	1.6%
SERC Reliability Corporation	SERC	53.1%	45.9%	1.0%
Southwest Power Pool	SPP	53.0%	44.8%	2.2%
Texas Regional Entity	TRE	35.5%	64.4%	0.1%
Western Electricity Coordinating Council	WECC	29.2%	70.7%	0.1%
U.S. Non-Baseload	U.S.	47.2%	51.2%	1.6%

Note: Data for Non-Baseload resources for NERC regions and the U.S. are from US EPA[1]. The data are from the 2017 version of eGRID, which use 2014 data.

Supplementary Table 10: Non-Baseload Weighted Fuel Mix by eGRID subregion

EPA eGRID Subregion	EPA Subregion Acronym	Coal	Natural Gas	Oil
ASCC Alaska Grid	AKGD	13.0%	74.7%	12.2%
ASCC Miscellaneous	AKMS	0.0%	57.7%	42.3%
WECC Southwest	AZNM	19.2%	80.8%	0.0%
WECC California	CAMX	0.9%	99.0%	0.1%
ERCOT All	ERCT	24.1%	75.7%	0.1%
FRCC All	FRCC	22.2%	76.5%	1.3%
HICC Miscellaneous	HIMS	2.9%	0.0%	97.1%
HICC Oahu	HIOA	0.0%	0.0%	100.0%
MRO East	MROE	79.5%	18.5%	2.0%
MRO West	MROW	82.8%	16.7%	0.5%
NPCC New England	NEWE	14.1%	80.0%	6.0%
WECC Northwest	NWPP	54.0%	45.8%	0.3%
NPCC NYC/Westchester	NYCW	0.0%	96.1%	3.9%
NPCC Long Island	NYLI	0.0%	88.0%	12.0%
NPCC Upstate NY	NYUP	22.7%	74.9%	2.4%
RFC East	RFCE	40.6%	57.3%	2.1%
RFC Michigan	RFCM	71.8%	26.9%	1.4%
RFC West	RFCW	80.9%	17.6%	1.5%
WECC Rockies	RMPA	62.7%	37.3%	0.0%
SPP North	SPNO	82.5%	17.3%	0.2%
SPP South	SPSO	51.7%	45.9%	2.4%
SERC Mississippi Valley	SRMV	33.5%	64.7%	1.8%
SERC Midwest	SRMW	93.3%	6.6%	0.1%
SERC South	SRSO	50.0%	49.6%	0.4%
SERC Tennessee Valley	SRTV	70.7%	28.8%	0.5%
SERC Virginia/Carolina	SRVC	47.3%	50.9%	1.8%

Note: Data for Non-Baseload Emissions factors for EPA eGRID subregions are from US EPA[1].

Supplementary Table 11: Non-Baseload Life Cycle Electricity Emissions by NERC Region

NERC Region	Acronym	Upstream GHGs [g CO_2e/kWh]	Direct GHGs [g CO_2e/kWh]	Life Cycle GHGs [g CO_2e/kWh]
Alaska Systems Coordinating Council	ASCC	100	423	523
Florida Reliability Coordinating Council	FRCC	91	531	622
Hawaiian Islands Coordinating Council	HICC	148	594	742
Midwest Reliability Organization	MRO	78	551	629
Northeast Power Coordinating Council	NPCC	95	443	538
Reliability First Corporation	RFC	81	773	854
SERC Reliability Corporation	SERC	84	649	733
Southwest Power Pool	SPP	85	639	724
Texas Regional Entity	TRE	88	551	639
Western Electricity Coordinating Council	WECC	89	327	416
U.S. Non-Baseload	U.S.	86	568	654
Future Low-Carbon Grid Scenario				200

Note: Data for Non-Baseload resources for NERC regions and the U.S. are from US EPA[1]. The data are from the 2017 version of eGRID, which use 2014 data.

Supplementary Table 12: Alternative Non-Baseload Life Cycle Emissions Factors for Electricity for eGRID Subregions

EPA eGRID Subregion	EPA Subregion Acronym	Upstream GHGs [g CO_2e/kWh]	Direct GHGs [g CO_2e/kWh]	Life Cycle GHGs [g CO_2e/kWh]
ASCC Alaska Grid	AKGD	99	412	511
ASCC Miscellaneous	AKMS	118	459	577
WECC Southwest	AZNM	91	429	520
WECC California	CAMX	95	272	367
ERCOT All	ERCT	90	485	575
FRCC All	FRCC	91	532	623
HICC Miscellaneous	HIMS	147	472	619
HICC Oahu	HIOA	149	662	811
MRO East	MROE	79	780	860
MRO West	MROW	78	503	581
NPCC New England	NEWE	95	430	526
WECC Northwest	NWPP	84	264	348
NPCC NYC/Westchester	NYCW	97	567	664
NPCC Long Island	NYLI	102	608	710
NPCC Upstate NY	NYUP	92	364	455
RFC East	RFCE	88	632	720
RFC Michigan	RFCM	81	782	863
RFC West	RFCW	79	822	901
WECC Rockies	RMPA	82	605	687
SPP North	SPNO	78	689	767
SPP South	SPSO	85	673	758
SERC Mississippi Valley	SRMV	89	608	697
SERC Midwest	SRMW	75	837	913
SERC South	SRSO	85	591	676
SERC Tennessee Valley	SRTV	80	759	839
SERC Virginia/Carolina	SRVC	86	583	669
Future Low-Carbon Grid Scenario				200

Note: Data for Non-Baseload Emissions factors for EPA eGRID subregions are from US EPA[1]. The data are from the 2017 version of eGRID, which use 2014 data. CH4 and N2O global warming potentials from IPCC AR5[31] are used. Future low-carbon assumption represents sensitivity case for low-carbon regional electricity grid.

Supplementary Table 13: Life cycle g GHGs per package for small drones

	Small Drone with 200 g GHG/kWh electricity	Small Drone in California	Small Drone U.S. Average	Small Drone in Missouri	Small Drone with 1000 g GHG/kWh electricity
**Battery Production**	16	16	16	16	16
**Transportation Electricity**	16	30	53	74	81
**Warehouse Electricity**	140	257	458	639	700
**Warehouse Natural Gas**	118	118	118	118	118
**Total**	290	421	645	847	915

Supplementary Table 14: Life cycle g GHGs per package for large drones

	Large Drone with 200 g GHG/kWh electricity	Large Drone in California	Large Drone U.S. Average	Large Drone in Missouri	Large Drone with 1000 g GHG/kWh electricity
**Battery Production**	158	158	158	158	158
**Transportation Electricity**	162	297	530	740	810
**Warehouse Electricity**	140	257	458	639	700
**Warehouse Natural Gas**	118	118	118	118	118
**Total**	578	830	1264	1654	1786

Supplementary Table 15: Life cycle g GHGs per package for parcel delivery trucks

	Diesel Truck	Natural Gas Truck	Electric Truck in California	Electric Truck U.S. Average	Electric Truck in Missouri
**Battery Production**			40	40	40
**Transportation Electricity**			345	615	857
**Upstream Transportation Fuels**	138	112			
**Transportation Fuels Combustion**	590	471			
**Warehouse Electricity**	229	229	129	229	320
**Warehouse Natural Gas**	59	59	59	59	59
**Total**	1015	871	572	943	1276

Supplementary Table 16: Life cycle g GHGs per package for parcel delivery vans

	Gasoline Van	Electric Van in California	Electric Van U.S. Average	Electric Van in Missouri
**Battery Production**		11	11	11
**Transportation Electricity**		104	185	258
**Upstream Transportation Fuels**	83			
**Transportation Fuels Combustion**	213			
**Warehouse Electricity**	458	257	458	639
**Warehouse Natural Gas**	118	118	118	118
**Total**	872	489	772	1026

Supplementary Table 17: Life cycle g GHGs per package for passenger cars

	Personal Gasoline Car	Personal Electric Vehicle in California	Personal Electric Vehicle U.S. Average	Personal Electric Vehicle in Missouri
**Battery Production**		205	205	205
**Transportation Electricity**		1619	2887	4027
**Upstream Transportation Fuels**	1280			
**Transportation Fuels Combustion**	3288			
**Warehouse Electricity**		128	229	320
**Warehouse Natural Gas**	59	59	59	59
**Total**	4627	2011	3380	4611

Supplementary References

1. US EPA. *Emissions & Generation Resource Integrated Database, eGRID2010 Version 1.0, Ninth Edition*. (U.S. Environmental Protection Agency, 2014).
2. IEA. *CO2 Emissions from Fuel Combustion*. (International Energy Agency, 2015).
3. Kopera, J. J. Inside the Nickel metal hydride battery. *Cobasys MI USA* (2004). Available at: http://www.cobasys.com/pdf/tutorial/inside_nimh_battery_technology.pdf

4. Energizer Battery Manufacturing Inc. Product Datasheet ENERGIZER NH15-2300. Form No. EBC - 7102WB.
5. Beck, F. & Rüetschi, P. Rechargeable batteries with aqueous electrolytes. *Electrochimica Acta* **45,** 2467–2482 (2000). Available at: http://data.energizer.com/PDFs/nh15-2300.pdf
6. Power Genix. Cell Type PGX AA Consumer. (2009). Available at: http://www.battex.info/special.php?file_id=48
7. Parker, J. F., Chervin, C. N., Nelson, E. S., Rolison, D. R. & Long, J. W. Wiring zinc in three dimensions re-writes battery performance-dendrite-free cycling. *Energy Environ. Sci.* **7,** 1117–1124 (2014).
8. PolyPlus Battery Company Inc. Advanced Lithium Battery Technology. (2009). Available at: http://www.polyplus.com/technology.html
9. Jian, Z. *et al.* Core–Shell-Structured CNT@RuO2 Composite as a High-Performance Cathode Catalyst for Rechargeable Li–O2 Batteries. *Angew. Chem. Int. Ed.* **53,** 442–446 (2014).
10. Shim, J., Striebel, K. A. & Cairns, E. J. The Lithium/Sulfur Rechargeable Cell: Effects of Electrode Composition and Solvent on Cell Performance. *J. Electrochem. Soc.* **149,** A1321–A1325 (2002).
11. Scrosati, B. & Garche, J. Lithium batteries: Status, prospects and future. *J. Power Sources* **195,** 2419–2430 (2010).
12. Hyperion. Hydperion G3 LiPo - Specifications. Available at: http://media.hyperion.hk/dn/g3lipo/G3-Specs-EN.pdf
13. Howard, W. F. & Spotnitz, R. M. Theoretical evaluation of high-energy lithium metal phosphate cathode materials in Li-ion batteries. *J. Power Sources* **165,** 887–891 (2007).

14. E-One Moli Energy Corp. Molicel Lithium-ion Rechargable Battery, Product Data Sheet, Model ICR18650M. Available at: http://www.molicel.com/hq/product/DM_ICR18650M-V1-80072.pdf
15. E-One Moli Energy Corp. Molicel Lithium-ion Rechargable Battery, Product Data Sheet, Model IHR18650C. Available at: http://www.molicel.com/tw/product/DM_IHR18650C-V1-80073.pdf
16. A123 Systems Inc. Nanophosphate Lithium Ion Prismatic Pouch Cell AMP20M1HD-A, MD100105-03. (2012). Available at: http://wamtechnik.pl/files/specs/793.pdf
17. Larminie, J., Dicks, A. & McDonald, M. S. *Fuel cell systems explained*. **2,** (Wiley New York, 2003).
18. Chau, K. T., Wong, Y. S. & Chan, C. C. An overview of energy sources for electric vehicles. *Energy Convers. Manag.* **40,** 1021–1039 (1999).
19. Hartman, J. *Nitrous Oxide Performance Handbook*. (MotorBooks International, 2009).
20. Fuentes, M. J., Font, R., Gómez-Rico, M. F. & Martín-Gullón, I. Pyrolysis and combustion of waste lubricant oil from diesel cars: Decomposition and pollutants. *J. Anal. Appl. Pyrolysis* **79,** 215–226 (2007).
21. Rabis, A., Rodriguez, P. & Schmidt, T. J. Electrocatalysis for Polymer Electrolyte Fuel Cells: Recent Achievements and Future Challenges. *Acs Catal.* **2,** 864–890 (2012).
22. Ramsden, T., Ulsh, M., Sprik, S., Kurtz, J. & Ainscough, C. *Direct Methanol Fuel Cell Material Handling Equipment Deployment*. (NREL, 2012).
23. Toyota Motor Corporation. Toyota's New, More Efficient Engines. (2014). Available at: http://newsroom.toyota.co.jp/en/download/2610289
24. United Farmers of Alberta. *Lubricant Handbook*. (2008). Available at: http://www.ufa.com/PDFFiles/lubricantHandbook/Complete%20Lube%20Handbook.pdf

25. Department of Energy. *Fuel Cell Technologies Office Multi-Year Research, Development, and Demonstration Plan - Section 3.3 Hydrogen Storage*. (Office of Energy Efficiency and Renewable Energy, 2012).

24. Toshiba. Toshiba launches direct methanol fuel cell in Japan as external power for mobile devices. (2009). Available at: http://www.toshiba.com/taec/news/press_releases/2009/dmfc_09_580.jsp

25. Horizon Fuel Cell Technologies. H-12 Fuel Cell Stack User Manual. (2011). Available at: http://media.wix.com/ugd/047f54_20cb233d0d0cfde250aae8035191421b.pdf

28. Tong, F., Jaramillo, P. & Azevedo, I. M. L. Comparison of Life Cycle Greenhouse Gases from Natural Gas Pathways for Medium and Heavy-Duty Vehicles. *Environ. Sci. Technol.* (2015). doi:10.1021/es5052759

29. US DOE. FuelEconomy.gov. (2016). Available at: http://fueleconomy.gov/. (Accessed: 22nd September 2016)

30. Argonne National Laboratory. *GREET.Net Database*. (U.S. Department of Energy, 2014).

31. IPCC. *Climate Change 2013: The Physical Science Basis. Contribution of Working Group I to the Fifth Assessment Report of the Intergovernmental Panel on Climate Change [Stocker, T.F., D. Qin, G.-K. Plattner, M. Tignor, S.K. Allen, J. Boschung, A. Nauels, Y. Xia, V. Bex and P.M. Midgley (eds.)]*. (Cambridge University Press, 2013).
